# *TaSPL14-7A* is a conserved regulator controlling plant architecture and yield traits in common wheat (*Triticum aestivum* L.)

**DOI:** 10.3389/fpls.2023.1178624

**Published:** 2023-04-05

**Authors:** Lina Cao, Tian Li, Shuaifeng Geng, Yinhui Zhang, Yuxue Pan, Xueyong Zhang, Fang Wang, Chenyang Hao

**Affiliations:** ^1^ College of Agronomy, Gansu Agricultural University, Lanzhou, China; ^2^ Key Laboratory of Crop Gene Resources and Germplasm Enhancement, Institute of Crop Sciences, Chinese Academy of Agricultural Sciences, Beijing, China; ^3^ Gansu Provincial Key Laboratory of Aridland Crop Science, Gansu Agricultural University, Lanzhou, China; ^4^ Gansu Key Laboratory of Crop Improvement & Germplasm Enhancement, Gansu Agricultural University, Lanzhou, China

**Keywords:** *TaSPL14-7A*, tiller number, kernel weight, haplotype analysis, wheat

## Abstract

Plant architecture is a crucial influencing factor of wheat yield and adaptation. In this study, we cloned and characterized *TaSPL14*, a homologous gene of the rice ideal plant architecture gene *OsSPL14* in wheat. *TaSPL14* homoeologs (*TaSPL14-7A*, *TaSPL14-7B* and *TaSPL14-7D*) exhibited similar expression patterns, and they were all preferentially expressed in stems at the elongation stage and in young spikes. Moreover, the expression level of *TaSPL14-7A* was higher than that of *TaSPL14-7B* and *TaSPL14-7D*. Overexpression of *TaSPL14-7A* in wheat resulted in significant changes in plant architecture and yield traits, including decreased tiller number and increased kernel size and weight. Three *TaSPL14-7A* haplotypes were identified in Chinese wheat core collection, and haplotype-based association analysis showed that *TaSPL14-7A-Hap1/2* were significantly correlated with fewer tillers, larger kernels and higher kernel weights in modern cultivars. The haplotype effect resulted from a difference in *TaSPL14-7A* expression levels among genotypes, with *TaSPL14-7A-Hap1/2* leading to higher expression levels than *TaSPL14-7A-Hap3*. As favorable haplotypes, *TaSPL14-7A-Hap1/2* underwent positive selection during global wheat breeding over the last century. Together, the findings of our study provide insight into the function and genetic effects of *TaSPL14* and provide a useful molecular marker for wheat breeding.

## Introduction

SPL (SQUAMOSA-PROMOTER BINDING PROTEIN-LIKE) is a type of plant-specific transcription factor. The SPL family is characterized by a highly conserved SBP (SQUAMOSA-PROMOTER BINDING PROTEIN) domain consisting of approximately 76 amino acid residues and the presence of a zinc-binding domain containing two zinc-binding sites, and a conserved nuclear localization signal at the C-terminus (Yamasaki et al., 2004; Birkenbihl et al., 2005). This gene family was found to be widespread and evolutionarily conserved in plants (Preston and Hileman, 2013; Wang and Wang, 2015). There were 17 *SPL* genes in *Arabidopsis* (Yang et al., 2008), 31 in maize (Hultquist and Dorweiler, 2008), 19 in rice (Xie et al., 2006), and 56 in wheat (Zhu et al., 2020). Interestingly, many plant *SPL* genes possess the recognition target sites of miRNA156/157, and their expression is fine-tuned by miRNA156/157 at the transcriptional and posttranscriptional levels, generating an elaborative regulatory module during plant evolution (Gandikota et al., 2007). Increasing evidence indicates that *SPL* genes play an important role in plant growth and development, including leaf initiation (Wang and Li, 2008), shoot maturation (Shikata et al., 2009), fruit ripening (Manning et al., 2006), cell division and grain filling (Wang et al., 2012), and stress response and hormone signal transduction (Stone et al., 2005; Riese et al., 2008; Wang et al., 2009).

In rice, the *IPA1* (*Ideal Plant Architecture 1*) quantitative trait locus encodes OsSPL14 and has great value in rice yield improvement. A point mutation (C-to-A) in *OsSPL14* disrupts the OsmiR156-directed cleavage of its mRNA, thereby increasing *OsSPL14* transcription and resulting in ideal plant architecture traits for rice, such as reduced tiller number and increased lodging resistance and grain yield (Jiao et al., 2010). Furthermore, another epigenetic allele (*ipa1-2D*) also exhibits elevated expression of *OsSPL14* due to the presence of tandem repeat sequences in the promoter region, which is associated with reduced DNA methylation at the *IPA1* promoter, thus alleviating the epigenetic repression of *IPA1* (Zhang et al., 2017). This, in turn, results in a similar ideal plant architecture and enhanced yield. More interestingly, deleting a 54-bp cis-regulatory region in *IPA1 via* a tiling-deletion-based CRISPR-Cas9 screen resolves the trade-off between tiller number and grains per panicle, leading to substantially enhanced grain yield per plant (Song et al., 2022). The deleted fragment is a target site for An-1 transcription factor binding to inhibit *IPA1* expression in panicles and roots, offering a realistic solution for optimizing the expression levels of *IPA1* with a less deleterious effect on over-reduction of tiller number. Thus, *OsSPL14* plays an important role in regulating plant architecture and yield, and fine-tuning its expression level is key to achieving high yield in rice.

Plant architecture is a key determinant of crop productivity and adaptability. It is a complex trait involving multiple factors, such as plant height, branching pattern, and leaf shape (Wang et al., 2018; Hang et al., 2021). Common wheat (*Triticum aestivum* L., AABBDD) is a major staple crop and an important human food source. Optimizing plant architecture is an important goal of wheat breeding programs to improve varieties and increase yields. Recently, some key genes that control plant architecture and yield have been identified in wheat. *TaCol-B5*, which encodes a CONSTANS-like transcription factor, can modify spike architecture and enhance grain yield in wheat. Overexpression of *TaCol-B5* in wheat increases the numbers of spikelet nodes per spike, tillers, and spikes, thereby increasing grain yield by 11.9% under field conditions (Zhang et al., 2022a). Moreover, the miR319/TaGAMYB3 module also controls plant architecture and grain yield in wheat. Repressing miR319 or increasing *TaGAMYB3* expression can result in favorable plant architecture traits and enhance grain yield in field plot tests (Jian et al., 2022). Therefore, more important regulators of plant architecture and yield need to be identified and further studied in wheat.

In this study, we isolated and characterized three homoeologs of *TaSPL14* from wheat group 7 chromosomes. The expression patterns of *TaSPL14* homoeologs were investigated at various developmental stages in wheat. Overexpression of *TaSPL14-7A* in wheat led to a reduction in tillers as well as increases in kernel size and weight. We analyzed sequence variations in *TaSPL14-7A* among wheat accessions and performed an association analysis between phenotypes and haplotypes. *TaSPL14-7A-Hap1/2* was associated with multiple traits in modern cultivars, including lower tiller number, larger kernel size, and higher kernel weight. More importantly, *TaSPL14-7A-Hap1/2* underwent positive selection during global wheat breeding over the last century. Our findings provide evidence that *TaSPL14* is a conserved regulator of plant architecture and yield traits and provide a functional marker for molecular marker-assisted selection in wheat breeding.

## Materials and methods

### Plant materials and phenotype assessment

A total of 505 Chinese wheat accessions, including 157 landraces and 348 modern cultivars, were used for marker screening and association analyses (Liu et al., 2020). Agronomic trait data for these wheat accessions were collected from plants grown in three environments: Luoyang (112°26′E, 34°37′N), Henan Province, China, in 2002 and 2005, and Shunyi (116°65′W, 40°13′N), Beijing, China, in 2010. In addition, a total of 1,051 global wheat accessions, including 384 European, 480 North American, 83 former USSR, 53 CIMMYT, and 51 Australian modern wheat cultivars, were used to investigate the global distribution of *TaSPL14-7A* haplotypes. For spatiotemporal expression analysis, different tissue samples were collected from Chinese Spring (CS) wheat plants at different developmental stages. *TaSPL14* transgenic and wild-type wheat were grown in a greenhouse under long-day conditions (16 h light/8 h darkness) at 23°C. For each T_3_-generation transgenic line, the phenotypes of at least 10 plants were analyzed.

### RNA extraction and qRT-PCR analysis

Total RNA was extracted from various Chinese Spring tissues using Fruit-mate™ for RNA Purification (Takara Biomedical Technology, Beijing, China). cDNA was synthesized using the FastKing RT Kit (Tiangen Biotech) according to the manufacturer’s instructions. The coding sequences (CDSs) of *TaSPL14* homoeologs were cloned from Chinese Spring cDNA according to the Ensemblplants database (http://plants.ensembl.org/index.html). Genome-specific primers of *TaSPL14* homoeologs were designed using Primer Premier 5.0 software (http://www.premierbiosoft.com/) and validated by sequencing. Quantitative real-time PCR (qRT-PCR) assays were performed on a LightCycler 96 Real-Time PCR system (Roche Applied Science, Penzberg, Germany) using SYBR Premix Ex Taq (Takara Bio, Beijing, China). The wheat *Actin* gene was used as the internal reference (Liu et al., 2020). The relative expression of each gene was calculated according to the comparative CT method. All assays were performed three times in independent experiments. The primers used in this study were listed in **Supplementary Table 1**.

### Subcellular localization

The full-length CDS of *TaSPL14-7A* was amplified from a Chinese Spring cDNA sample and subcloned into the pCAMBIA1300-GFP vector. The derived construct was transformed into *Agrobacterium tumefaciens* strain GV3101 and then co-infiltrated into tobacco (*Nicotiana benthamiana*) leaves. After a 48 h of incubation, the transformed tobacco leaves were stained with 4′,6-diamidino-2-phenylindole (DAPI), and then GFP and DAPI fluorescence were both observed using an LSM880 laser scanning confocal microscope (Carl Zeiss, Jena, Germany).

### Vector construction and plant transformation

To construct the *TaSPL14-7A*-overexpression vector for wheat transformation, the full-length CDS of *TaSPL14-7A* was amplified and then cloned into a modified pCAMBIA3301 vector under the control of the maize *Ubiquitin* promoter as previously described (Zhang et al., 2022b). The resulting construct was mobilized into *Agrobacterium tumefaciens* strain EHA105 and then transformed into immature embryos of wheat cultivar Fielder *via* an Agrobacterium-mediated transformation method (Wang et al., 2017).

### *TaSPL14-7A* haplotype discovery and association analysis

A total of 36 wheat accessions (13 modern cultivars and 23 landraces) were initially used for detection of *TaSPL14-7A* sequence variations (**Supplementary Table 2**). Specific primers were designed to amplify the 2.5-kb promoter region and the 4.5-kb coding region of *TaSPL14-7A*. The resulting DNA fragments were sequenced. Sequence variations, including single nucleotide polymorphisms (SNPs) and insertion/deletion (InDels), were identified using DNASTAR (http://www.dnastar.com/). The NEBcutter V3.0 (https://nc3.neb.com/NEBcutter/) was used to query the restriction enzyme recognition sites, and two cleaved amplified polymorphic site (CAPS) markers of the *TaSPL14-7A* haplotypes were developed based on two SNPs (T/C at 144 bp and G/A at 4111 bp). Haplotype association analysis was performed based on a previously described method (Hou et al., 2014; Ma et al., 2016). In brief, the variance analyses were carried out using SPSS 16.0 (IBM Corporation). Phenotypic differences among haplotypes were determined using one-way ANOVA and Tukey’s test at a significance level of *P* < 0.05.

### Differential expression and promoter activity analysis of *TaSPL14-7A* haplotypes

A total of 15 wheat varieties were used for differential expression analysis of three *TaSPL14-7A* haplotypes (**Supplementary Table 3**). Total RNA was extracted from 1-cm young spikes and stems at the elongation stage of these wheat varieties and subjected to qRT-PCR assays. For promoter activity assays, the 2.5-kb promoter sequence of each *TaSPL14-7A* haplotype was amplified and cloned into the pGreenII 0800-LUC vector (Hellens et al., 2005). As described previously (Liu et al., 2020), the promoter activity of each haplotype was measured using the Dual-Luciferase Reporter Assay System (Promega, USA), and calculated according to the relative luciferase activity (LUC/REN ratio).

## Results

### Cloning and expression analysis of *TaSPL14* homoeologs in wheat

**Figure 1:**
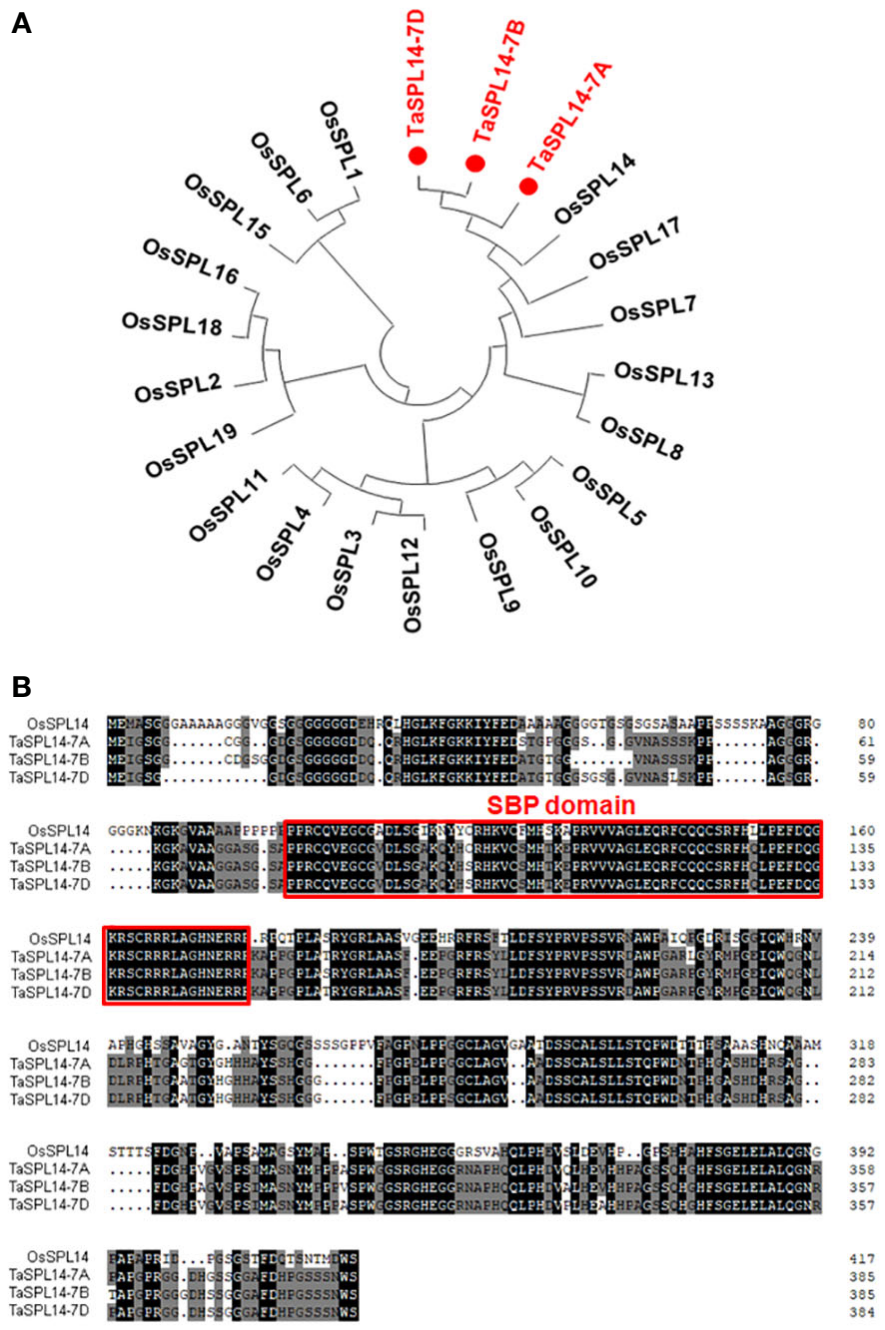
Phylogenetic tree and sequence alignment of TaSPL14 homoeologs. **(A)** Phylogenetic tree constructed from the 19 rice SPL proteins and three TaSPL14 homoeologs using the neighbor-joining method with a bootstrap value of 1000 by MEGA5.0. TaSPL14 homoeologs are marked in red. **(B)** Sequence alignment of OsSPL14 and TaSPL14 homoeologs. The conserved SBP domain is indicated by the red box.

To isolate *SPL14* homologous genes in wheat, the CDS of *OsSPL14* (Os08g0509600) was used as a query to search the Chinese Spring RefSeq v1.1 genome database (IWGSC, 2018). Three *SPL14* homoeologs located on chromosomes 7AS (*TraesCS7A02G246500*), 7BS (*TraesCS7B02G144900*), and 7DS (*TraesCS7D02G245200*) were obtained and designated *TaSPL14-7A*, *TaSPL14-7B*, and *TaSPL14-7D*, respectively. The phylogenetic analysis revealed that these three *TaSPL14* homoeologs have the closest homology to *OsSPL14* (**Figure 1A**). The genic regions of *TaSPL14-7A*, *TaSPL14-7B*, and *TaSPL14-7D* are 4,325, 3,817, and 3,863 bp in length, respectively, and each consists of 3 exons and 2 introns (**Supplementary Figure 1**). *TaSPL14-7A*, *TaSPL14-7B*, and *TaSPL14-7D* comprise 386, 386, and 385 amino acids, respectively, and share high sequence similarities with *OsSPL14* (~60%), especially in the conserved SBP domain (**Figure 1B**). These data suggest that *TaSPL14* homoeologs may have biological functions similar to those of *OsSPL14*.

**Figure 2:**
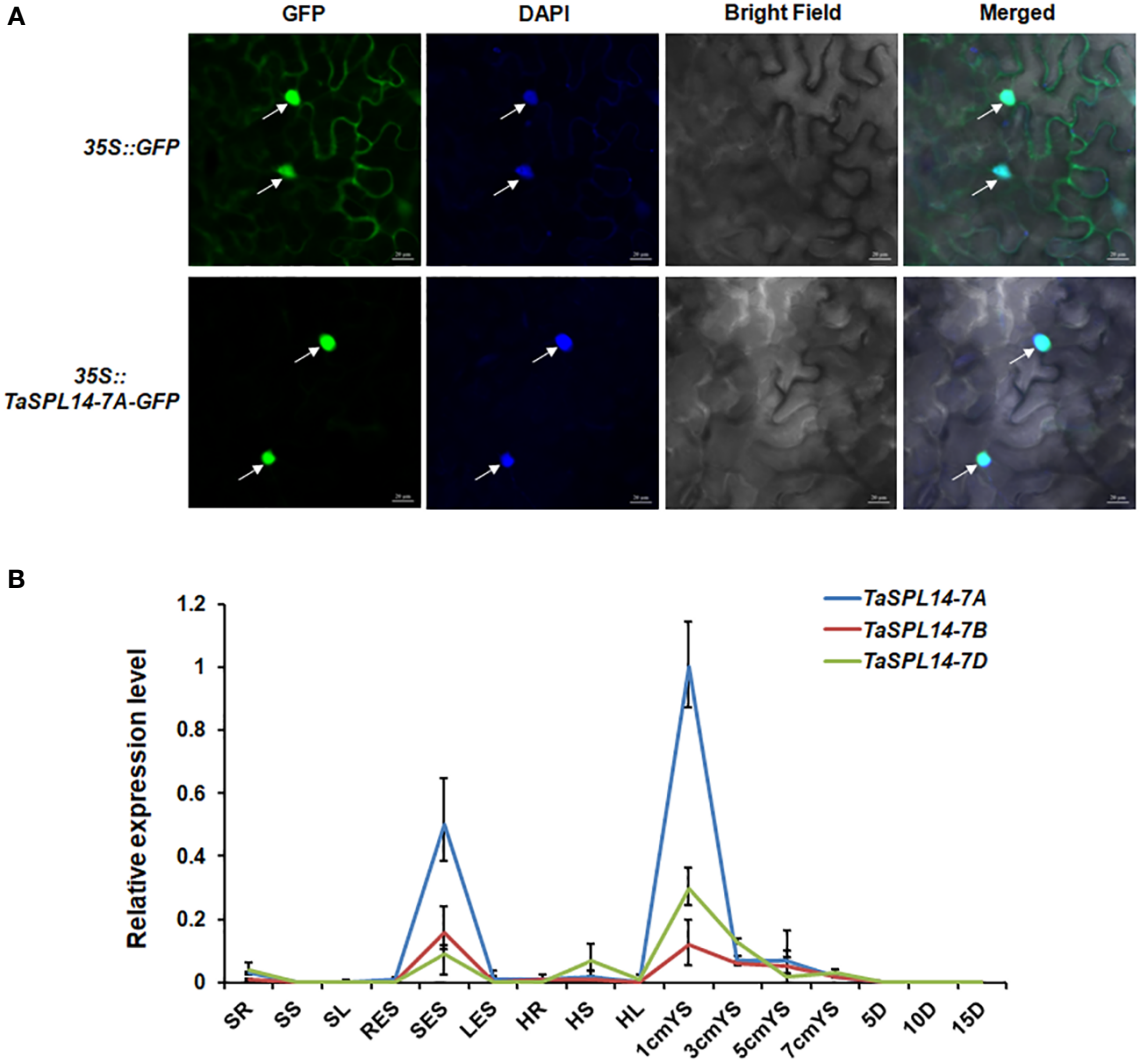
Characterization of TaSPL14 homoeologs. **(A)** Subcellular localization of TaSPL14-7A in tobacco leaves. GFP and TaSPL14-7A-GFP fusions under the control of the CaMV 35S promoter were transiently expressed in tobacco leaves. The arrows indicate nuclei stained with DAPI. Bar = 20 µm. **(B)** Spatial and temporal expression patterns of the three *TaSPL14* homoeologs in various Chinese Spring tissues. SR, seedling roots; SS, seedling stems; SL, seedling leaves; RES, roots at elongation stage; SES, stems at elongation stage; LES, leaves at elongation stage; HR, roots at heading stage; HS, stems at heading stage; HL, leaves at heading stage; 1cm–7cmYS, 1–7-cm young spikes; 5D–15D, grains at 5–15 days post-anthesis, respectively. Normalized values of *TaSPL14* expression relative to *Actin* were given as mean ± SD from three replicates.

To investigate the subcellular localization of *TaSPL14*, we transiently expressed the TaSPL14-7A-GFP (green fluorescent protein) fusion construct in tobacco (*Nicotiana benthamiana*) leaves. After 48 h of incubation and DAPI staining, the GFP and DAPI florescence signals were observed by laser confocal microscopy. As shown in **Figure 2A**, TaSPL14-7A-GFP fusion protein fluorescence was co-localized with DAPI-stained nucleus, whereas GFP fluorescence alone was present throughout the cell; therefore, *TaSPL14* is a nucleus-localized transcription factor similar to *OsSPL14*. Furthermore, genome-specific primers were used for qRT-PCR to explore the temporal and spatial expression of *TaSPL14* homoeologs. The results showed that the expression levels of *TaSPL14* homoeologs were significantly higher in stems at the elongation stage, and in young spikes (1 cm in length) and substantially lower in other tissues, including roots, leaves and grains (**Figure 2B**). Moreover, the expression level of *TaSPL14-7A* in all detected tissues was higher than that of *TaSPL14-7B* and *TaSPL14-7D*. Our results were consistent with the data in the wheat expression database (**Supplementary Figure 2**).

### Overexpression of *TaSPL14-7A* leads to reduced tiller number and increased kernel weight and size in wheat

**Figure 3:**
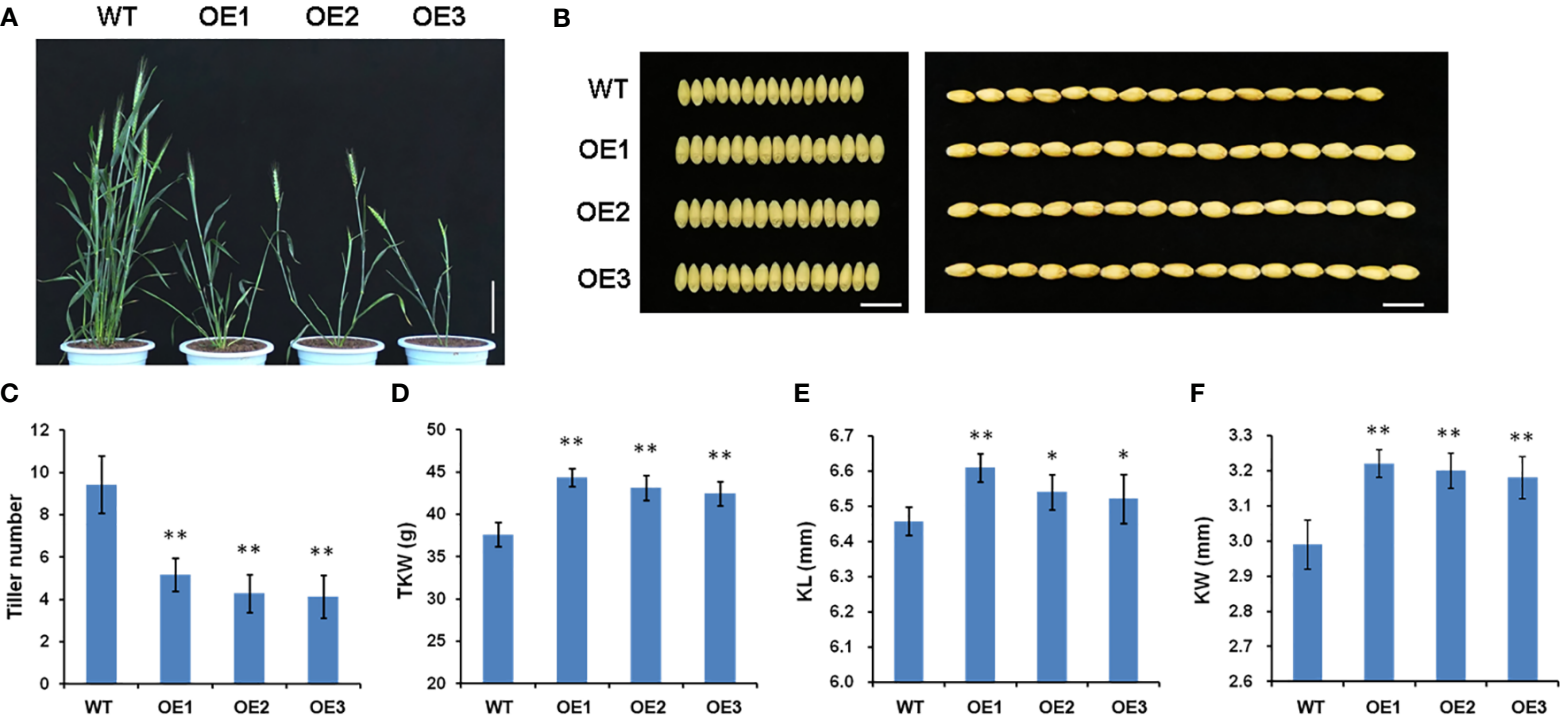
Phenotypic comparison of wild-type (WT) and three *TaSPL14-7A* overexpression (OE) lines. **(A)** Comparison of the plant architecture phenotype at heading stage among these genotypes. Bar = 20 cm. **(B)** Comparison of the kernel phenotypes among these genotypes. Bar = 1cm. **(C)** Tiller number; **(D)** Thousand kernel number (TKW); **(E)** Kernel length (KL); **(F)** Kernel width (KW). The values are presented as mean ± SD. ^**^ *P*<0.01; ^*^ *P*<0.05 (Student’s *t*-test).

To explore the physiological function of *TaSPL14* in wheat, we generated *TaSPL14-7A* overexpression lines in the hexaploid wheat cultivar Fielder using Agrobacterium-mediated transformation. The total expression levels of *TaSPL14* were examined in the transgenic plants using qRT-PCR, and the results showed that the *TaSPL14-7A* expression was significantly increased in all three representative overexpression (OE) lines (**Supplementary Figure 3**). Compared with the wild-type (WT) control, all the OE lines showed dramatic changes in plant architecture and yield traits (**Figures 3A, B**). The tiller number of OE lines was approximately 47.5%-56.1% less than that of the WT (**Figure 3C**). In contrast, OE lines had larger kernel size and weight, and the thousand-kernel weight (TKW) was 4.86 g-5.29 g higher than that of the WT (**Figure 3D**). Notably, the increased TKW in the transgenic plants mainly resulted from a 0.7%-2.2% increase in kernel length (KL) and a 6.4%-7.7% increase in kernel width (KW) (**Figures 3E, F**). Therefore, our phenotypic data clearly indicated that *TaSPL14* is a conserved pleiotropic regulator of plant architecture and yield traits, consistent with the phenotypic effects of *OsSPL14* (Jiao et al., 2010).

### *TaSPL14-7A* haplotypes are associated with tiller number and kernel weight and size

**Figure 4:**
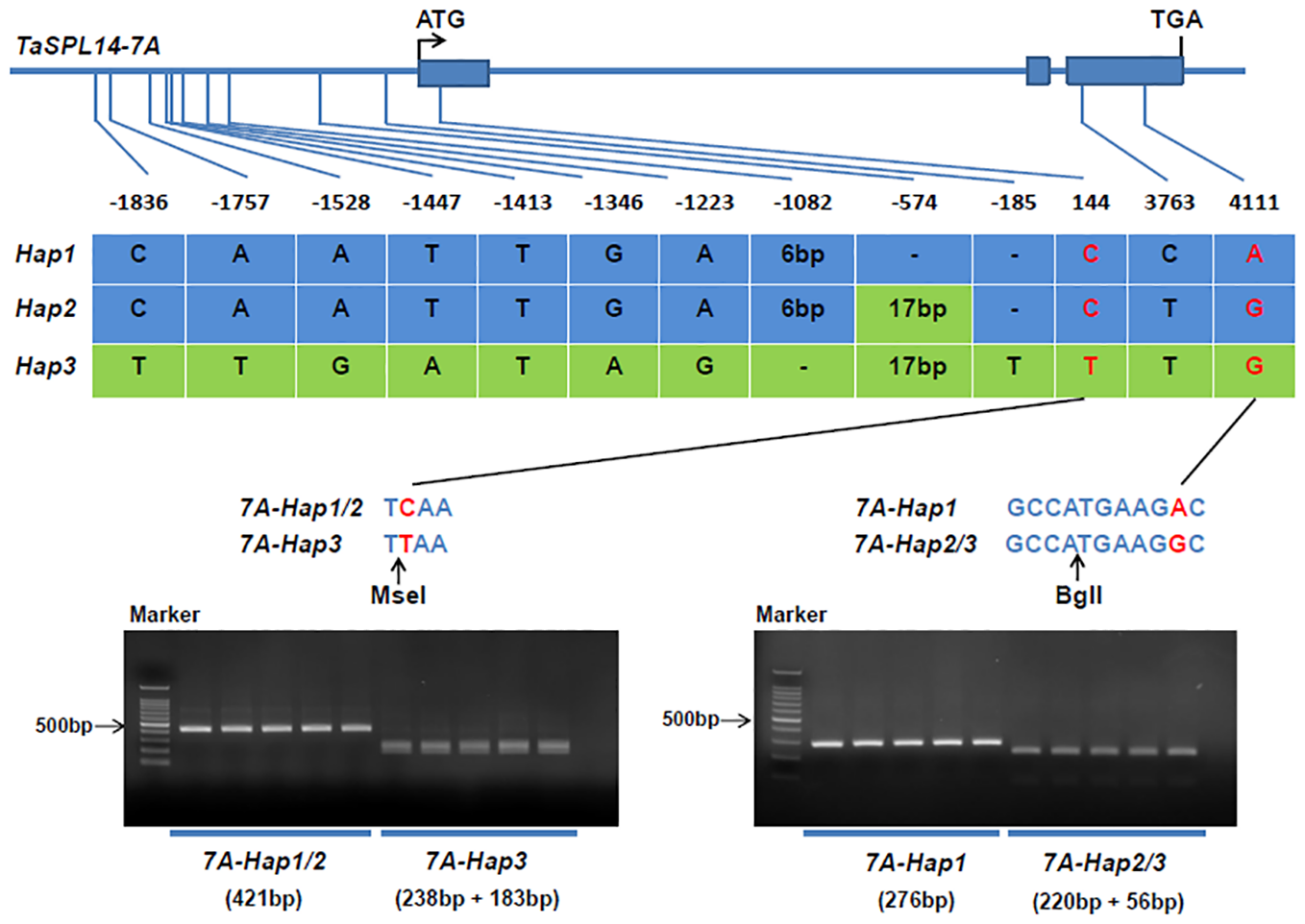
Identification of *TaSPL14-7A* haplotypes and development of molecular markers. The top panel shows a schematic diagram of the gene and 2.5 kb promoter structure of *TaSPL14-7A*. The ATG start codon was designated as position 1 bp. The bottom panel shows the polymorphic sites of *TaSPL14-7A*. A CAPS marker named CAPS-144 was developed based on the SNP (T/C) at 144 bp. Digestion of the amplified 421 bp fragment with MseI produced fragments of 238 bp and 183 bp for *TaSPL14-7A-Hap3* (T), whereas this fragment could not be digested for *TaSPL14-7A-Hap1/2* (C). A CAPS marker named CAPS-4111 was developed based on the SNP (G/A) at 4111 bp. Digestion of the amplified 276 bp fragment with BglI produced fragments of 220 bp and 56 bp for *TaSPL14-7A-Hap2/3* (G), whereas this fragment could not be digested for *TaSPL14-7A-Hap1* (A).

To detect natural variations in *TaSPL14* homoeologs, we re-sequenced the genome regions of 36 highly diversified wheat varieties, and the results showed that there were polymorphic loci in the genome regions of *TaSPL14-7A* and *TaSPL14-7B*, while no sequence differences were detected in *TaSPL14-7D*. Because *TaSPL14-7A* has the highest expression among *TaSPL14* homoeologs (**Figure 2B**), we developed two molecular markers, CAPS-144 and CAPS-4111, to distinguish the three *TaSPL14-7A* haplotypes (*TaSPL14-7A-Hap1/2/3*). CAPS-144 was developed based on the SNP (T/C) at the 144-bp position, which produced the recognition site of the restriction enzyme MseI at *TaSPL14-7A-Hap3*, but did not identify corresponding sites at *TaSPL14-7A-Hap1/2* (**Figure 4**). After two-step PCR amplification and enzyme digestion, the amplified fragments of *TaSPL14-7A-Hap3* were digested and separated (**Figure 4**). CAPS-4111 was developed based on the SNP (G/A) at the 4111-bp position, which produced the recognition sites of the restriction enzyme BglI at *TaSPL14-7A-Hap2/3* but did not identify the corresponding site at *TaSPL14-7A-Hap1* (**Figure 4**). After two-step PCR amplification and enzyme digestion, the amplified fragments of *TaSPL4-7A-Hap2/3* was digested and separated (**Figure 4**).

**Figure 5:**
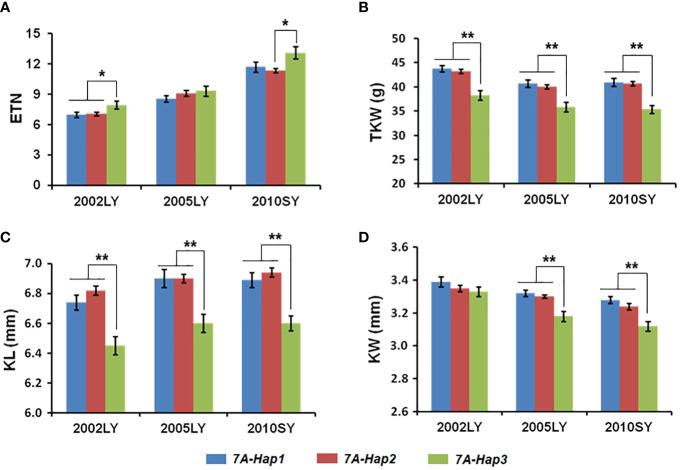
Association analysis of *TaSPL14-7A* haplotypes with tiller number and kernel traits in modern cultivars in three different environments. **(A)** Effective tiller number (ETN); **(B)** Thousand kernel weight (TKW); **(C)** Kernel length (KL); **(D)** Kernel width (KW) of modern cultivars with different *TaSPL14-7A* haplotypes. The x-axis represents different environments: Luoyang, 2002 (2002LY); Luoyang, 2005 (2005LY); Shunyi, 2010 (2010SY). The values are presented as mean ± SE. ^**^ *P*<0.01; ^*^ *P*<0.05 (ANOVA).

By using the newly developed molecular markers CAPS-144 and CAPS-4111, we genotyped 348 modern cultivars from Chinese wheat core collection (**Supplementary Table 4**). We then performed an association analysis between the *TaSPL14-7A* haplotypes and multiple agronomic traits measured in three environments (Luoyang, 2002; Luoyang, 2005; Shunyi, 2010). Significant differences in effective tiller number (ETN) were detected between *TaSPL14-7A-Hap1/2* and *TaSPL14-7A-Hap3* (**Figure 5A**; **Supplementary Table 5**). Moreover, the *TaSPL14-7A* haplotypes were significantly correlated with TKW, KL, and KW. The mean TKW of *TaSPL14-7A-Hap1/2* was 4.6 g-5.3 g higher than that of *TaSPL14-7A-Hap3* in all three environments (**Figure 5B**; **Supplementary Table 5**). Consistently, the mean KL and KW of *TaSPL14-7A-Hap1/2* were significantly higher than those of *TaSPL14-7A-Hap3* (**Figures 5C, D**; **Supplementary Table 5**). These results indicated that *TaSPL14-7A-Hap1/2* are favorable haplotypes associated with higher TKW and kernel size (KL and KW).

### Significant expression differences of *TaSPL14-7A* exist among haplotypes

**Figure 6:**
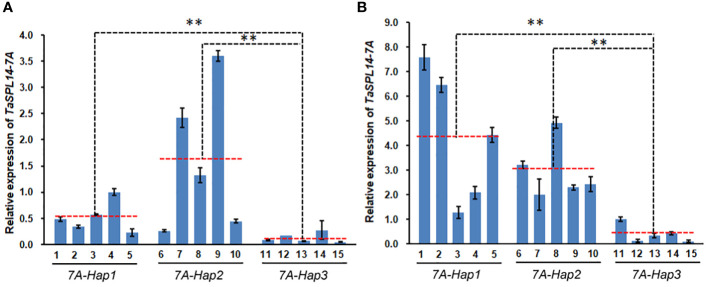
Differential expression of *TaSPL14-7A* in different haplotypes. **(A)** *TaSPL14-7A* expression was compared in the 1 cm young spikes. **(B)** *TaSPL14-7A* expression was compared in the stems at elongation stage. The red-dashed lines indicate the average expression levels of each haplotype accession. 1, Huoliaomai; 2, Dahongmai; 3, Baiyoumai; 4, Yangmai; 5, Dongnong 101; 6, Neimai 11; 7, Xiaohongpi; 8, Bihongsui; 9, Ganmai 46; 10, Jinchun 3; 11, Ganmai 6; 12, Xiangnong 3; 13, Xiaobaimai; 14, Xinkehan 9; 15, Lianglaiyou. ^**^ *P*<0.01 (ANOVA).

Most variations between *TaSPL14-7A-Hap1/2* and *TaSPL14-7A-Hap3* were located in the promoter regions and caused some changes in binding sites of important transcription factors such as WRKY, MYB and GRF (**Supplementary Figure 4A**). To test whether variations in the promoter region resulted in differential expression, we detected the expression of *TaSPL14-7A* in 1-cm young spikes and stems at the elongation stage in 15 wheat varieties with different haplotypes (**Supplementary Table 3**). As shown in **Figure 6**, *TaSPL14-7A* expression was significantly higher in the *TaSPL14-7A-Hap1/2* accessions than in the *TaSPL14-7A-Hap3* accessions in both 1-cm young spikes and stems, consistent with the hypothesis that *TaSPL14-7A-Hap1/2* with higher *TaSPL14-7A* expression is associated with lower tiller number and higher kernel weight and size. In addition, a minor difference in *TaSPL14-7A* expression was also observed between *TaSPL14-7A-Hap1* and *TaSPL14-7A-Hap2*, where *TaSPL14-7A-Hap2* accessions had the highest expression in 1-cm young spikes (**Figure 6A**), while *TaSPL14-7A-Hap1* accessions had the highest expression in stems (**Figure 6B**). In addition, we carried out promoter activity analysis through transient expression assay in *Nicotiana benthamiana* leaves. As shown in **Supplementary Figure 4B**, relative LUC activity (LUC/REN) of *TaSPL14-7A-Hap1* or *TaSPL14-7A-Hap2* promoter was about 2-fold greater than that of *TaSPL14-7A-Hap3* promoter, confirming that *TaSPL14-7A-Hap1/2* had higher promoter activity. These results suggest that *TaSPL14-7A-Hap1/2* are correlated with significantly increased expression levels of *TaSPL14-7A* due to their higher promoter activity.

### Geographic distribution and frequency change of *TaSPL14-7A* haplotypes in global wheat breeding

**Figure 7:**
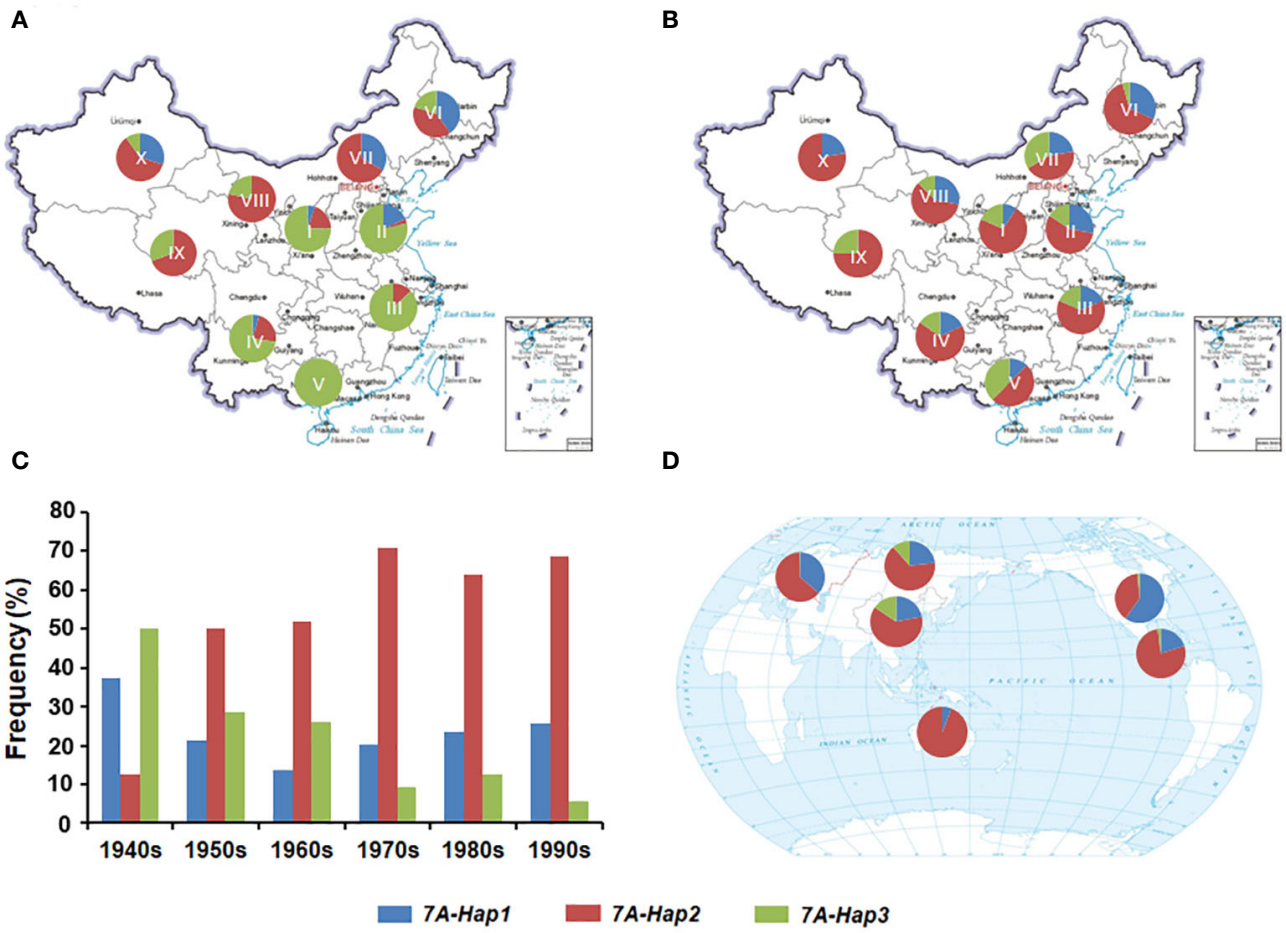
Geographic distribution and frequency change of *TaSPL14-7A* haplotypes. **(A)** Distribution of *TaSPL14-7A* haplotypes in Chinese landraces from ten agro-ecological zones (I, northern winter wheat region; II, Yellow and Huai River valley winter wheat region; III, low and middle Yangtze River valley winter wheat region; IV, southwestern winter wheat region; V, southern winter wheat region; VI, northeastern spring wheat region; VII, northern spring wheat region; VIII, northwestern spring wheat region; IX, Qinghai-Tibet spring-winter wheat region; X, Xinjiang winter-spring wheat region). **(B)** Distribution of *TaSPL14-7A* haplotypes in modern Chinese cultivars from ten agro-ecological zones. **(C)** Frequency changes of *TaSPL14-7A* haplotypes in different Chinese wheat breeding periods (from the 1940s to the 1990s). **(D)** Distribution of *TaSPL14-7A* haplotypes in major global wheat regions including China, North America, Europe, former USSR, CIMMYT, and Australia.

Previous studies have shown that favorable haplotypes can be positively selected and accumulate during the wheat breeding (Paux et al., 2012). To determine the geographic distribution and artificial selection of different *TaSPL14-7A* haplotypes, we genotyped 157 landraces and 348 modern cultivars from ten wheat agro-ecological zones in China. From landraces to modern cultivars, the proportions of *TaSPL14-7A-Hap1/2* were significantly increased in the major production zones (I-IV), indicating a strong positive selection in the wheat breeding process (**Figures 7A, B**). From the 1940s to 1990s, the frequency of favorable *TaSPL14-7A-Hap2* increased from 12.5% to 68.5%, whereas the frequency of *TaSPL14-7A-Hap3* gradually declined from 49.5% to 4.5% (**Figure 7C**). Therefore, favorable *TaSPL14-7A* haplotypes were subjected to strong selection pressure and became the dominant haplotypes in the modern cultivars.

Next, we performed a genotyping analysis of 1,051 modern cultivars from Europe, North America, the former USSR, CIMMYT, and Australia to assess the global geographic distribution pattern of the *TaSPL14-7A* haplotypes (**Supplementary Table 6**). Similar to the selection trend in China, the proportions of *TaSPL14-7A-Hap1/2* were also very high in the other five regions; especially in Australia, CIMMYT, Europe, and North America (**Figure 7D**). In these regions, the frequency of *TaSPL14-7A-Hap3* was very low, with the haplotype nearly eliminated. These results provide strong evidence that *TaSPL14-7A-Hap1/2* underwent positive selection as part of global wheat breeding.

## Discussion

The SPL gene has a highly conserved DNA-binding domain (SBP domain), which represents a family of plant-specific transcription factors and plays a key role in regulating plant development in various plant species, such as *Arabidopsis*, rice, and maize (Xie et al., 2006; Hultquist and Dorweiler, 2008; Yang et al., 2008). *IPA1* (*OsSPL14*) is a core regulator of plant architecture and yield, having pleiotropic effects on multiple important yield traits, including tiller number, panicle primary branch number and TKW (Jiao et al., 2010). In the present study, we isolated *OsSPL14* homologues in wheat through a bioinformatics approach. *TaSPL14* was identified as a close homologue of *OsSPL14*, and its homoeologs are located on chromosomes 7A, 7B, and 7D (**Figure 1A**). *TaSPL14* shares high sequence similarities with *OsSPL14* (~60%), particularly in the SBP protein domain (**Figure 1B**), suggesting that they have similar protein structures and functions. Furthermore, overexpression of *TaSPL14-7A* in common wheat cultivars resulted in significant changes in plant architecture and yield traits, such as a smaller tiller number and increased kernel weight and size (**Figure 3**), showing a phenotype similar to that of *IPA1* (*OsSPL14*) in rice (Jiao et al., 2010). Therefore, *TaSPL14* is a conserved negative regulator of tiller number, as well as a positive regulator of kernel weight and size. It should be noted that constitutive overexpression of *TaSPL14* cannot lead to an increase in grain yield because *TaSPL14* has opposite effects on tiller number and TKW, two key components of grain yield. Previous studies also showed that *IPA1* (*OsSPL14*) in rice regulates tiller number, stem diameter, and panicle primary branch number in a dose-dependent manner, but overexpression has a negative effect on grain yield (Zhang et al., 2017). These findings offer a realistic solution for achieving high-yield goals by optimizing the expression levels of *TaSPL14*. It is possible to balance tiller number and TKW by fine-tuning the tissue-specific expression of *TaSPL14*, e.g., weak alleles for the up-regulation of *TaSPL14* expression or gene editing of its cis-regulatory region.

In crops, variations in the coding regions account for only a small fraction of the large number of natural variations, many of which are deleterious and will be eliminated during evolution and selection due to their large impacts on protein functions. Unlike deleterious variations, most natural variations with phenotypic benefits are located in regulatory regions such as promoters and introns. In this study, we detected natural variations in *TaSPL14-7A* and found that most variations were located in the promoter region, including SNPs and InDels (**Figure 4**). Although three exonic SNPs were identified in the coding region, they did not cause obvious genetic effects according to haplotype-based association analysis results (**Figure 5**). In contrast, variations in the promoter region could result in differential expression of *TASPL14-7A* and significant alteration of promoter activity between *TaSPL14-7A-Hap1/2* and *TaSPL14-7A-Hap3*, providing supporting evidence for phenotypic differences and genetic effects of *TaSPL14-7A* haplotypes (**Figure 6**; **Supplementary Figure 4**). Similar regulatory patterns have been observed in many other yield-related genes in wheat, including *TaGW2-6A*, *TaGW2-6B*, *TaBT1-6B*, and *TaDA1-2A* (Su et al., 2011; Qin et al., 2014; Wang et al., 2019; Liu et al., 2020). Therefore, our present and previous studies supported that variations in regulatory regions are conducive to the rapid adaptation of crops and can allow gene expression to adapt to environmental changes. Therefore, these variations located in promoter regions can be used as potential target sites for wheat breeding and selection in the future.

Marker-assisted selection (MAS) is an efficient method for crop genetic improvement. The accumulation of favorable alleles or haplotypes is mainly involved in wheat breeding, so MAS can be used to significantly accelerate the breeding process (Gupta et al., 2023). In this study, we developed two CAPS markers to distinguish three *TaSPL14-7A* haplotypes, and the association analysis indicated that the *TaSPL14-7A* haplotypes were significantly correlated with tiller number, TKW, kernel length, and kernel width in modern cultivars (**Figure 5**), among which *TaSPL14-7A-Hap1/2* were favorable haplotypes correlated with a smaller tiller number, higher TKW, and larger kernel size. According to the breeding process of three haplotypes in 10 different agro-ecological zones of China, *TaSPL14-7A-Hap2* was subjected to strong positive selection from landraces to modern cultivars (**Figures 7A, B**). Moreover, the frequency of *TaSPL14-7A-Hap2* increased significantly from the 1940s to 1990s in China (**Figure 7C**). As favorable haplotypes, *TaSPL14-7A-Hap1/2* had become dominant in the global modern cultivars (**Figure 7D**). Interestingly, *TaSPL14-7A-Hap1/2* have a strong positive effect on TKW but a weak negative effect on tiller number based on haplotype association analysis (**Figure 5**), possibly representing a superior plant architecture in modern wheat cultivar improvement. Balancing the tiller number and TKW is critical for achieving higher grain yield potential in wheat breeding. Therefore, *TaSPL14-7A-Hap1/2* and their functional markers will be applied in future wheat yield improvement.

## Conclusion

In this study, we characterized *TaSPL14-7A* as a negative regulator of tiller number and a positive regulator of kernel weight and size in wheat. Haplotype analysis indicated that the favorable *TaSPL14-7A* haplotypes were associated with superior plant architecture and yield traits and underwent positive selection during global wheat breeding. Our findings provide insight into the genetic effects of *TaSPL14* and its potential application in wheat improvement.

## Data availability statement

The original contributions presented in the study are included in the article/**Supplementary Material**. Further inquiries can be directed to the corresponding author.

## Author contributions

TL, FW and CH planned and designed the research. LC, TL, YZ, and YP performed the experiments. TL, SG, XZ, FW and CH collected and analyzed the data. LC, TL, and CH wrote the manuscript.
