# Supplementary material for: TaSPL14-7A is a conserved regulator controlling plant architecture and yield traits in common wheat (Triticum aestivum L.)
Source: Front Plant Sci. 2023 Apr 5;14:1178624. doi: 10.3389/fpls.2023.1178624 (PMC10113487; doi:10.3389/fpls.2023.1178624)
Supplement: Supplementary file 1 [file DataSheet_1.pdf]

Supplementary Material

1 Supplementary Figures and Tables

1.1 Supplementary Figures

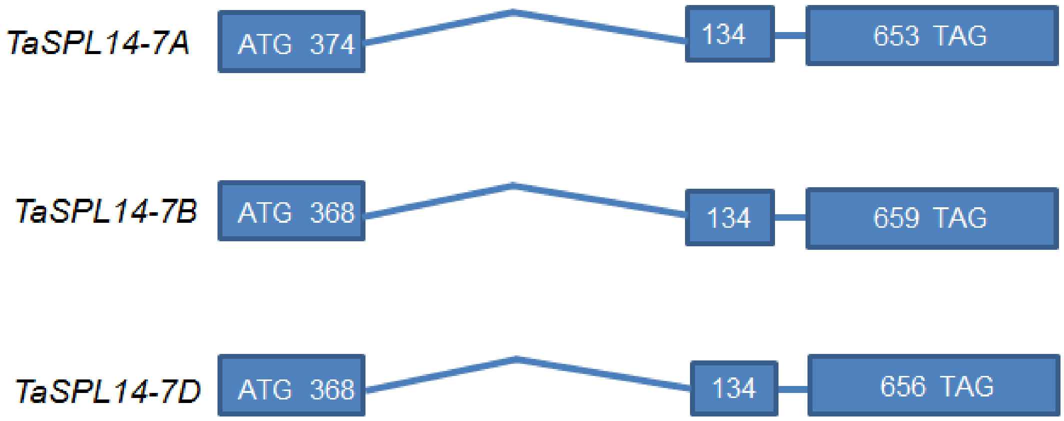

Supplementary Figure 1. Gene structures of the *TaSPL14* homoeologs. The blue rectangles represent exons, the lines between exons represent introns, and the number indicates the length of exons in base pairs.

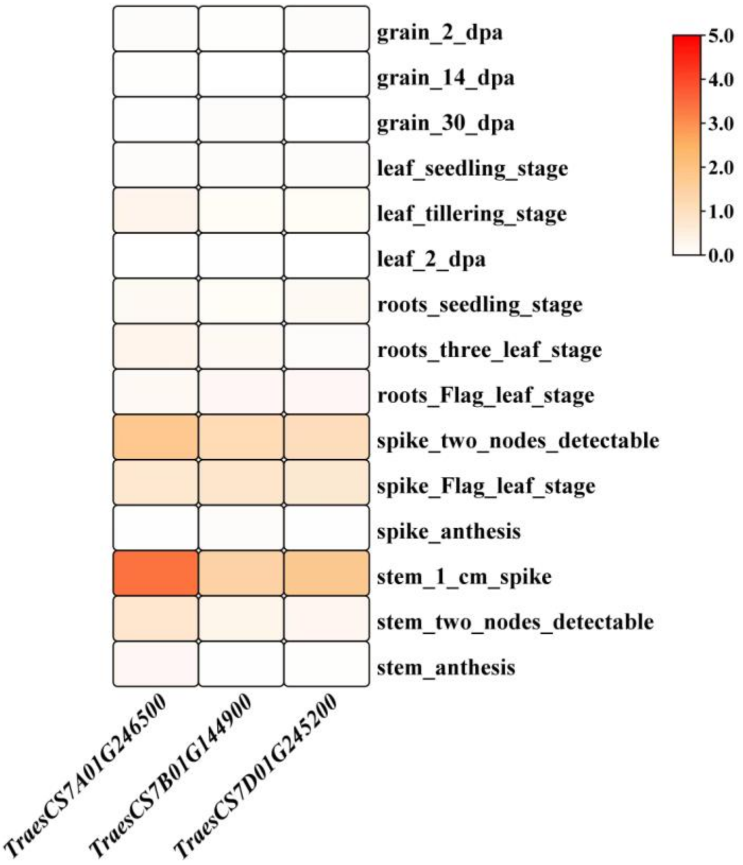

Supplementary Figure 2. Heat map of expression profiles of *TaSPL14* homoeologs. The tissue expression data of *TaSPL14* homoeologs are obtained from the wheat expression database (http://www.wheat–expression.com/). Expression levels of *TaSPL14* homoeologs are presented according to the color scale, in which red color and white color represent high and low transcript abundance respectively. *TaSPL14-7A*, *TraesCS7A02G246500*; *TaSPL14-7B*, *TraesCS7B02G144900*; *TaSPL14-7D*, *TraesCS7D02G245200*.

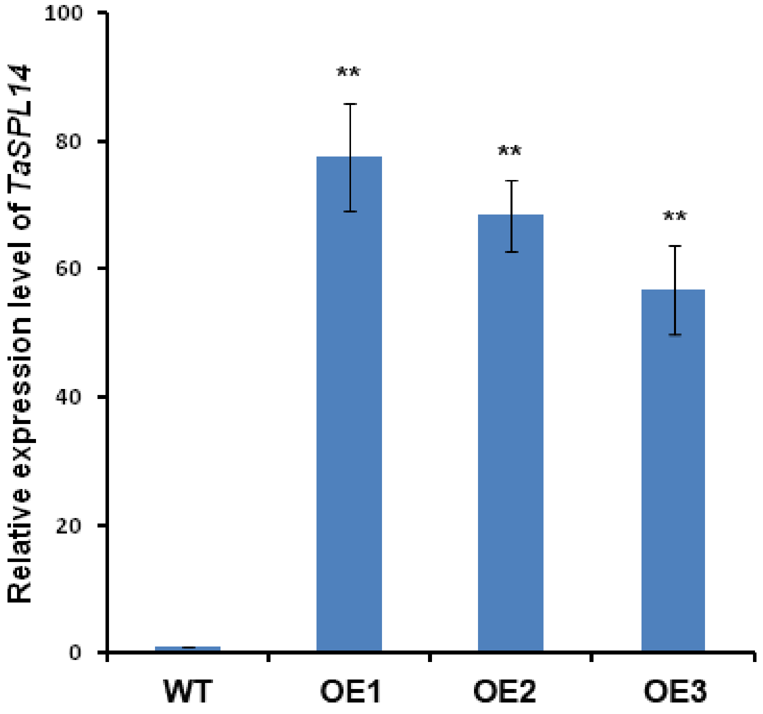

Supplementary Figure 3. Relative expression level of *TaSPL14* in seedling leaves of wild type (WT) and three representative T_3 generation overexpression (OE) lines (OE-1–3). The values are presented as mean ± SD. $^{**}P < 0.01$ (ANOVA) indicates a significant difference to the WT.

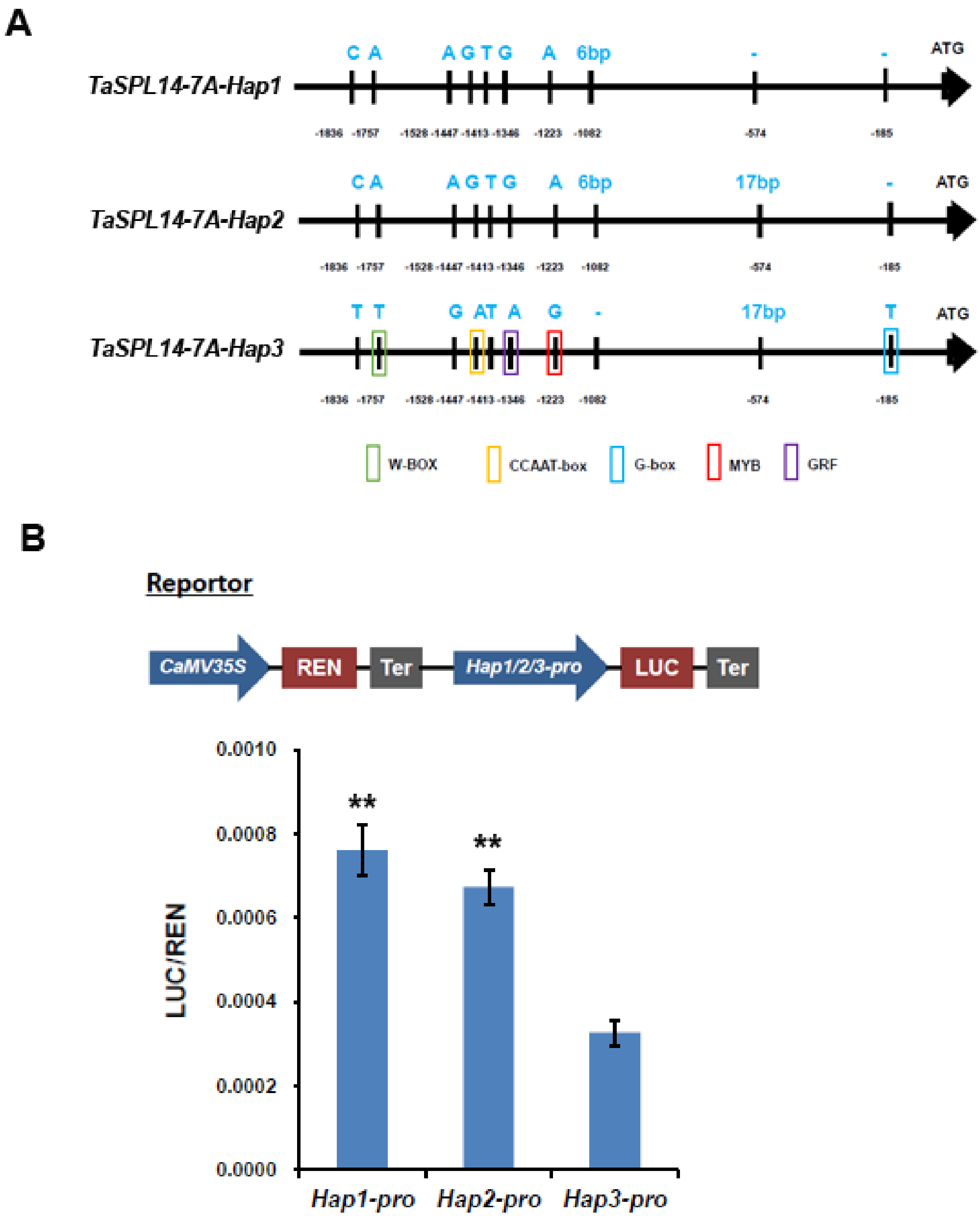

Supplementary Figure 4. Cis-elements prediction and promoter activity analysis of *TaSPL14-7A* haplotypes. (A) Predicted cis-elements in the promoter regions of *TaSPL14-7A*. Three haplotypes are formed by 7 SNPs and 2 InDels in the *TaSPL14-7A* promoter regions. Different cis-elements are shown in boxes with different colors. W-Box (TTGAC**Y**): WRKY transcription factor binding motif; CCAAT-box (CCA**A**T): CCAAT transcription factor binding motif; G-box (C**T**GGTC): light responsive element; MYB (AGC**G**G): MYB transcription factor binding motif; GRF (TTTTCAG**A**): GRF transcription factor binding motif. (B) Promoter activity assays of *TaSPL14-7A* haplotypes. The promoter of each haplotype is cloned into the pGreenII 0800-LUC vector, and the promoter activity is measured according to the LUC/REN ratio. The values are presented as mean ± SD. $^{**}P < 0.01$ (ANOVA) indicates a significant difference to the *Hap3-pro*.

1.2 Supplementary Tables

Supplementary Table 1. Primers used in this study.

Primers used for gene cloning and vector construction (5′-3′)	
TaSPL14-OE-F	GTCGACTCTAGAGGATCCATGGAGATTGGAAGCGG
TaSPL14-OE-R	GCTCTCTAGAACTAGTCTACAGAGACCAGTTGGACG
Primers used for qRT-PCR (5′-3′)	
TaSPL14-7A-qRT-F	TCGCTGCATCCTTTGAAGAA
TaSPL14-7A-qRT-R	CTTGCCACTGGATTTCACCA
TaSPL14-7B-qRT-F	TCGCTGCATCCTTTGAAGAA
TaSPL14-7B-qRT-R	TGAGGACGCAGGTCTAGGTTC
TaSPL14-7D-qRT-F	TCGCTGCATCCTTTGAAGAA
TaSPL14-7D-qRT-R	TTGGAGGGAGCTCTGGGC
TaActin-F	AGGTGCCCTGAGGTGCTGTT
TaActin-R	GCCAAAATAGAGCCACCGAT
Primers used for molecular marker development	
**CAPS144**	
TaSPL14-A-F1	GCCGCACGGCAGAGGAT
TaSPL14-A-R1	GAAGCGGAAGGCCCCAC
TaSPL14- A-F2	CAGCAAGCAGGCAAGAGCA
TaSPL14-A-R2	GCGGGGCTCCTTGGTGTGC
**CAPS4111**	
TaSPL14 A-F3	TGTATTCCAAACTCTACTGAAA
TaSPL14-A-R3	GGTGTGCTTGTGGTCTCTTAA
TaSPL14- A-F4	TCCCTCCAGGTGGGTGTCT
TaSPL14-A-R4	GCACCTCGTGGAGCTGGAC

Supplementary Table 2. The 36 wheat accessions used for polymorphism discovery.

Number	Accession	Haplotype	Variety type
1	Dingxingzhai	*TaSPL14-7A-Hap2*	Landrace
2	Hongpidongmai	*TaSPL14-7A-Hap3*	Landrace
3	Sanyuehuang	*TaSPL14-7A-Hap3*	Landrace
4	Baihuamai	*TaSPL14-7A-Hap3*	Landrace
5	Huomai	*TaSPL14-7A-Hap3*	Landrace
6	Biantouguangkemai	*TaSPL14-7A-Hap3*	Landrace
7	Shanxibaimai	*TaSPL14-7A-Hap3*	Landrace
8	Baimazha	*TaSPL14-7A-Hap3*	Landrace
9	Baimangmai	*TaSPL14-7A-Hap3*	Landrace
10	Zhumaoyuanzitou	*TaSPL14-7A-Hap3*	Landrace
11	Hongmangzi	*TaSPL14-7A-Hap2*	Landrace
12	Chanbuzhi	*TaSPL14-7A-Hap3*	Landrace
13	Wangshuibai	*TaSPL14-7A-Hap3*	Landrace
14	Dabaimai	*TaSPL14-7A-Hap3*	Landrace
15	Dalibanmang	*TaSPL14-7A-Hap3*	Landrace
16	Jinhuangmai	*TaSPL14-7A-Hap2*	Landrace
17	Zijiehong	*TaSPL14-7A-Hap2*	Landrace
18	Xiaobaimang	*TaSPL14-7A-Hap2*	Landrace
19	Dahongmai	*TaSPL14-7A-Hap1*	Landrace
20	Sankecun	*TaSPL14-7A-Hap2*	Landrace
21	Dabaipi	*TaSPL14-7A-Hap1*	Landrace
22	Honglidangnianlao	*TaSPL14-7A-Hap1*	Landrace
23	Paozimai	*TaSPL14-7A-Hap2*	Landrace
24	jingyang 60	*TaSPL14-7A-Hap3*	Modern cultivar
25	Fuzhuang 30	*TaSPL14-7A-Hap3*	Modern cultivar
26	Xinong 6028	*TaSPL14-7A-Hap3*	Modern cultivar
27	Shite14	*TaSPL14-7A-Hap1*	Modern cultivar
28	Zhemai 1	*TaSPL14-7A-Hap3*	Modern cultivar
29	Huzhuhong	*TaSPL14-7A-Hap2*	Modern cultivar
30	Mingxian169	*TaSPL14-7A-Hap3*	Modern cultivar
31	Dingxi24	*TaSPL14-7A-Hap1*	Modern cultivar
32	Nongda 139	*TaSPL14-7A-Hap2*	Modern cultivar
33	Shijiazhuang 54	*TaSPL14-7A-Hap1*	Modern cultivar
34	Zhongyou 9507	*TaSPL14-7A-Hap2*	Modern cultivar
35	Jinghong 5	*TaSPL14-7A-Hap1*	Modern cultivar
36	Lumai1	*TaSPL14-7A-Hap1*	Modern cultivar

Supplementary Table 3. The 15 wheat accessions used for differential expression analysis of *TaSPL14-7A* haplotypes.

Number	Accession	Haplotype	Variety type
1	Huoliaomai	*TaSPL14-7A-Hap1*	Landrace
2	Dahongmai	*TaSPL14-7A-Hap1*	Landrace
3	Baiyoumai	*TaSPL14-7A-Hap1*	Landrace
4	Yangmai	*TaSPL14-7A-Hap1*	Landrace
5	Dongnong 101	*TaSPL14-7A-Hap1*	Modern cultivar
6	Neimai 11	*TaSPL14-7A-Hap2*	Modern cultivar
7	Xiaohongpi	*TaSPL14-7A-Hap2*	Landrace
8	Bihongsui	*TaSPL14-7A-Hap2*	Modern cultivar
9	Ganmai 46	*TaSPL14-7A-Hap2*	Modern cultivar
10	Jinchun 3	*TaSPL14-7A-Hap2*	Modern cultivar
11	Ganmai 6	*TaSPL14-7A-Hap3*	Modern cultivar
12	Xiangnong 3	*TaSPL14-7A-Hap3*	Modern cultivar
13	Xiaobaimai	*TaSPL14-7A-Hap3*	Landrace
14	Xinkehan 9	*TaSPL14-7A-Hap3*	Modern cultivar
15	Lianglaiyou	*TaSPL14-7A-Hap3*	Modern cultivar

Supplementary Table 4. Genotypes of 157 landraces and 348 modern cultivars in Chinese wheat core collections.

Number	Accession	*TaSPL14-7A* haplotype	Agro-ecological zones
**Landraces**			
1	Xiaobaimai	*Hap2*	VII
2	Hongpixiaomai	*Hap2*	VII
3	Dabaipi	*Hap1*	VII
4	Xiaohongpi	*Hap2*	VII
5	Dingxingzhai	*Hap2*	VII
6	Honglidangnianlao	*Hap2*	VII
7	Chunxiaomai	*Hap2*	VII
8	Huoliaomai	*Hap1*	VII
9	Dahongmai	*Hap1*	VII
10	Shanxibaimai	*Hap3*	I
11	Niuzhijia	*Hap3*	I
12	Mahuaban	*Hap3*	I
13	Jiahongmai	*Hap3*	I
14	Hongjinmai	*Hap3*	I
15	Baiqimai	*Hap3*	I
16	Xiaokouhong	*Hap3*	I
17	Lanhuamai	*Hap3*	I
18	Daimanghongmai	*Hap3*	I
19	Zhuoludongmai	*Hap2*	I
20	Hongmai	*Hap3*	I
21	Honglaomai	*Hap2*	I
22	Youmangbaifu	*Hap3*	I
23	Hongpidongmai	*Hap3*	I
24	Panshiwumang	*Hap3*	I
25	Youmangbaifu	*Hap3*	I
26	Baiqiumai	*Hap3*	I
27	Laomai	*Hap2*	I
28	Xiaobaimang	*Hap2*	I
29	Xianmai	*Hap3*	III
30	Jiangxizao	*Hap3*	III
31	Honghuazao	*Hap3*	III
32	Jiangdongmen	*Hap3*	III
33	Dahuangpi	*Hap2*	III
34	Chongyanghongmai 1	*Hap3*	III
35	Zaowutian	*Hap3*	III
36	Liuzhutou	*Hap3*	III
37	Chanbuzhi	*Hap3*	III
38	Zhumaoyuanzitou	*Hap3*	III
39	Shuilizhan	*Hap3*	III

40	Huangshuibai	*Hap3*	III
41	Baipu	*Hap3*	III
42	Zaoxiaomai	*Hap3*	III
43	Lanxizaoxiaomai	*Hap3*	III
44	Wangshuibai	*Hap3*	III
45	Wuyuanmai	*Hap3*	III
46	Chejianzi	*Hap3*	III
47	Heshangmai	*Hap3*	III
48	Nuomai	*Hap3*	III
49	Mangxiaomai	*Hap3*	III
50	Sankecun	*Hap2*	III
51	Paozimai	*Hap2*	III
52	Baiyoumai	*Hap1*	VI
53	Yangmai	*Hap1*	VI
54	Donghuachunmai	*Hap3*	VI
55	Huoqiu	*Hap2*	VI
56	Daqingmang	*Hap2*	VI
57	Guangtou	*Hap2*	VI
58	Chaoanxiaomai	*Hap3*	V
59	Chike	*Hap3*	V
60	Songruimai	*Hap3*	V
61	Shengen	*Hap3*	V
62	Shanglinxiaomai	*Hap3*	V
63	Baimangmai	*Hap3*	II
64	Huangguaxian	*Hap3*	II
65	Banjiemang	*Hap3*	II
66	Laolaixia	*Hap3*	II
67	Louguding	*Hap3*	II
68	Xishanbiansui	*Hap3*	II
69	Honggoudou	*Hap3*	II
70	Baihuomai	*Hap3*	II
71	Sanyuehuang	*Hap3*	II
72	Hongqiangchang	*Hap3*	II
73	Youzimai	*Hap1*	II
74	Pingyuan 50	*Hap1*	II
75	Baibiansui	*Hap1*	II
76	Baiqimai	*Hap3*	II
77	Baituzitou	*Hap3*	II
78	Youmangsaogudan	*Hap3*	II
79	Buyanghong	*Hap3*	II
80	Mazhamai	*Hap3*	II
81	Qiangchangmai	*Hap1*	II
82	Huomai	*Hap3*	II
83	Meiqianwu	*Hap3*	II

84	Jiangmai	*Hap3*	II
85	Sanyuehuang	*Hap3*	II
86	Xiaofoshou	*Hap1*	II
87	Hongheshangtou	*Hap3*	II
88	Dakoumai	*Hap3*	II
89	Tumangmai	*Hap3*	II
90	Baitiaoyu	*Hap3*	II
91	Baimangmai	*Hap3*	II
92	Dayuhua	*Hap3*	II
93	Fumai	*Hap1*	II
94	Laoqimai	*Hap1*	II
95	Chushanbao	*Hap3*	II
96	Zijiehong	*Hap2*	II
97	Dalibanmang	*Hap3*	II
98	Liuyuehuang	*Hap2*	IX
99	Gejiaxiang	*Hap2*	IX
100	Geerhongmai	*Hap2*	IX
101	Dachunbaisilengmai	*Hap2*	IX
102	Bailanghuimai	*Hap3*	IX
103	Bendihuanghuamai	*Hap2*	IX
104	Zhahong	*Hap2*	IX
105	Motuoxiaomai	*Hap3*	IX
106	Bianbachunmai 6	*Hap3*	IX
107	Baimangxiaomai	*Hap3*	IX
108	Wujiangzhuo	*Hap2*	IX
109	Muzongzhuoga	*Hap2*	IX
110	Kangdingxiaomai	*Hap2*	IX
111	Shanmai	*Hap2*	VIII
112	Yizhimai	*Hap3*	VIII
113	Dabaimai	*Hap3*	VIII
114	Galaohan	*Hap2*	VIII
115	Huoliyan	*Hap2*	VIII
116	Shanmai	*Hap2*	VIII
117	Hongtuzi	*Hap2*	VIII
118	Baidatou	*Hap2*	VIII
119	Huangjinmai	*Hap2*	VIII
120	Hongmangmai	*Hap2*	VIII
121	Dabaimai	*Hap2*	VIII
122	Baiqitou	*Hap2*	VIII
123	Baimazha	*Hap3*	VIII
124	Laotutou	*Hap2*	VIII
125	Tongjiabaxiaomai	*Hap3*	IV
126	Honghuamai	*Hap3*	IV
127	Baimaizi	*Hap3*	IV

128	Chengduguangtou	*Hap3*	IV
129	Jiangmai	*Hap3*	IV
130	Baihuamai	*Hap3*	IV
131	Huanxiangguo	*Hap3*	IV
132	Hanzhongbai	*Hap3*	IV
133	Xiaosanyuehuang	*Hap3*	IV
134	Suotiaohongmai	*Hap3*	IV
135	Hongxumai	*Hap3*	IV
136	Zipi	*Hap3*	IV
137	Baimangmai	*Hap3*	IV
138	Hongmangzi	*Hap2*	IV
139	Yuqiumai	*Hap1*	IV
140	Yangmai	*Hap2*	IV
141	Yangmai	*Hap2*	IV
142	Zhushimai	*Hap2*	IV
143	biantouguangkemai	*Hap3*	IV
144	Changmangshibiantou	*Hap3*	IV
145	Zhugoumai	*Hap2*	IV
146	Dianxihongkeyangmai	*Hap3*	IV
147	Baidongmai	*Hap3*	X
148	Hongchunmai	*Hap1*	X
149	Chunmai	*Hap2*	X
150	Hongdongmai	*Hap1*	X
151	Hongdongmai	*Hap2*	X
152	Wumangchunmai	*Hap2*	X
153	Hongchunmai	*Hap1*	X
154	Hongjinbaoyin	*Hap2*	X
155	Hongdongmai	*Hap2*	X
156	Wumangchunmai	*Hap2*	X
157	Zhongguochun	*Hap3*	IV
**Modern cultivars**			
1	Dingxian 72	*Hap3*	I
2	Huabei 672	*Hap2*	I
3	Beijing 8	*Hap2*	I
4	Beijing 15	*Hap3*	I
5	Nongda 139	*Hap2*	I
6	Nongda 183	*Hap2*	I
7	Nongda 311	*Hap2*	I
8	Dongfanghong 3	*Hap2*	I
9	Keyi 23	*Hap3*	I
10	Jingnong 94-32	*Hap2*	I
11	Jingnong 81-49	*Hap2*	I
12	Jinghua 1	*Hap2*	I
13	Beinongda BL8	*Hap2*	I

14	Hebuyu 6068	*Hap1*	I
15	Jinghe 91-P19	*Hap2*	I
16	Jingnong 86-89	*Hap2*	I
17	Kecheng 1	*Hap2*	I
18	Xiaoshan 8	*Hap2*	I
19	Jingpin 12	*Hap1*	I
20	Pindong 904110-3	*Hap1*	I
21	Pinkang 244	*Hap2*	I
22	Yuandong 821	*Hap2*	I
23	Yuandong 822	*Hap1*	I
24	Fengkang 2	*Hap2*	I
25	Fengkang 8	*Hap2*	I
26	An 85 Zhong 124-1	*Hap2*	I
27	Qiaoliang BW41	*Hap3*	I
28	Zhongda 89-60192-2	*Hap2*	I
29	Beijing 8694	*Hap2*	I
30	Zhongyou 9507	*Hap2*	I
31	Lang 8302	*Hap2*	I
32	Jimai 17	*Hap2*	I
33	Tang 85-5032	*Hap2*	I
34	Tang 78042	*Hap2*	I
35	Henong 3	*Hap2*	I
36	Taifu 1	*Hap2*	I
37	Jinmai 8	*Hap2*	I
38	Jinmai 16	*Hap2*	I
39	Jinmai 31	*Hap2*	I
40	Jin 3052-5	*Hap2*	I
41	Jinzhong 103	*Hap2*	I
42	Changzhi 5557	*Hap2*	I
43	Changzhi 6406	*Hap3*	I
44	Lvhan 328	*Hap3*	I
45	Jinmai 11	*Hap2*	I
46	Taiyuan 351	*Hap2*	I
47	Jin 1410	*Hap1*	I
48	Mingxian 169	*Hap3*	I
49	Gongnong 4	*Hap3*	I
50	Taiyuan 2112	*Hap2*	I
51	Yanan 11	*Hap2*	I
52	Yanan 18	*Hap2*	I
53	Pingliang 30	*Hap3*	I
54	Xifeng 16	*Hap2*	I
55	Pingliang 32	*Hap2*	I
56	Qingshui 15-41(2)	*Hap3*	I
57	Shite 14	*Hap1*	II

58	Shijiazhuang 407	*Hap2*	II
59	Shijiazhuang 4	*Hap2*	II
60	Shijiazhuang 34	*Hap2*	II
61	Shijiazhuang 54	*Hap1*	II
62	Zaoxinshi	*Hap2*	II
63	Pin 39	*Hap2*	II
64	Jimai 23	*Hap1*	II
65	Shi 82-5201	*Hap1*	II
66	Cangzhou 1	*Hap2*	II
67	12040	*Hap2*	II
68	Hengshui 7004	*Hap2*	II
69	Hengdalihong	*Hap1*	II
70	Ji 93C6 156-2	*Hap2*	II
71	Jimai 20	*Hap2*	II
72	Gaobi-13	*Hap1*	II
73	Jimai 14	*Hap2*	II
74	Jimai 19	*Hap2*	II
75	Jishi 5032	*Hap1*	II
76	Han 4564	*Hap2*	II
77	1817	*Hap3*	II
78	Maijin 1	*Hap3*	II
79	Jinyang 1045	*Hap2*	II
80	Jinmai 20	*Hap2*	II
81	Jin 865096	*Hap2*	II
82	Jinmai 37	*Hap2*	II
83	Linyuan 129	*Hap1*	II
84	Pingyang 27	*Hap1*	II
85	Qida 195	*Hap2*	II
86	Jinan 2	*Hap2*	II
87	Jinan 9	*Hap2*	II
88	Youbao	*Hap2*	II
89	Huangtai 103	*Hap1*	II
90	Taishan 1	*Hap2*	II
91	Taishan 4	*Hap2*	II
92	Jinan 17	*Hap1*	II
93	Lumai 22	*Hap2*	II
94	Jimai 19	*Hap2*	II
95	Lumai 10	*Hap3*	II
96	Lainongpinxi 22	*Hap2*	II
97	Laizhou 953	*Hap2*	II
98	Lumai 1	*Hap1*	II
99	Lumai 5	*Hap2*	II
100	Shannong PH85-4	*Hap2*	II
101	Lunong 784081	*Hap2*	II

102	Lunong 86(5)174	*Hap1*	II
103	Luzi 0863169	*Hap2*	II
104	Luzi 0884142	*Hap2*	II
105	Taishan 7	*Hap1*	II
106	Teng 80-1-2	*Hap2*	II
107	Changwei 20	*Hap2*	II
108	Yanzhong 144	*Hap3*	II
109	Yannong 3	*Hap2*	II
110	Yannong 15	*Hap1*	II
111	Lumai 7	*Hap1*	II
112	Lumai 9	*Hap2*	II
113	Neixiang 5	*Hap1*	II
114	Anxuan 2	*Hap3*	II
115	Zhengzhou 6	*Hap3*	II
116	Bonong 7023	*Hap2*	II
117	Mengxian 2	*Hap2*	II
118	Wan 7107	*Hap1*	II
119	Yumai 18	*Hap1*	II
120	Yumai 54	*Hap1*	II
121	Bainong 3217	*Hap2*	II
122	Yumai 2	*Hap2*	II
123	Lankao 906	*Hap2*	II
124	Yuanzhu 55	*Hap3*	II
125	Zhengzhou 8761	*Hap2*	II
126	Yumai 14	*Hap3*	II
127	Zhengzhou 4	*Hap2*	II
128	Yu 7106-0-22-1-3-2B	*Hap1*	II
129	Zhengzhou 741	*Hap2*	II
130	Hua 852895-2	*Hap2*	II
131	Yu 30691-1-3	*Hap1*	II
132	Zheng 87305-0-13	*Hap1*	II
133	Zhengzi R84019-0-7-4-0-1	*Hap1*	II
134	Wenmai 6	*Hap2*	II
135	Yumai 7	*Hap2*	II
136	Anmai 95 Zhong 35	*Hap2*	II
137	Jingyang 60	*Hap3*	II
138	Bima 1	*Hap1*	II
139	Bima 4	*Hap3*	II
140	Shannong 9	*Hap3*	II
141	Xinong 6028	*Hap3*	II
142	Fengchan 3	*Hap3*	II
143	Jinguangmai	*Hap3*	II
144	Aifeng 3	*Hap1*	II
145	Xiaoyan 4	*Hap3*	II

146	Jingyang 30	*Hap3*	II
147	Baomai 3	*Hap2*	II
148	Baomai 5	*Hap1*	II
149	Qinmai 8	*Hap1*	II
150	Liken 2	*Hap2*	II
151	Shan 8786-0-3	*Hap3*	II
152	Shan 70-1	*Hap2*	II
153	Shannong 7859	*Hap2*	II
154	Shannong 229	*Hap2*	II
155	Fuzhuang 30	*Hap3*	II
156	Qinmai 3	*Hap2*	II
157	Fen 22	*Hap2*	II
158	Xiannong 39	*Hap2*	II
159	Xiannong 151	*Hap2*	II
160	Shan 7219-8-11-2-1	*Hap1*	II
161	Xiaoyan 6	*Hap2*	II
162	Gaoyou 503	*Hap2*	II
163	Ningfengmai	*Hap2*	II
164	Huaimai 11	*Hap1*	II
165	Huaimai 12	*Hap2*	II
166	Xuzhou 19	*Hap2*	II
167	Xuzhou 21	*Hap2*	II
168	Xuzhou 22	*Hap1*	II
169	Bu 84111	*Hap2*	II
170	Wo 80	*Hap2*	II
171	Xiaonong 76189	*Hap2*	II
172	Zhangdong 29	*Hap2*	II
173	Pingliang 35	*Hap1*	II
174	Wanyuan 28 Dali	*Hap2*	III
175	Anhui 11	*Hap2*	III
176	Anhui 3	*Hap1*	III
177	Mengfeng 8	*Hap2*	III
178	Anhui 9	*Hap2*	III
179	Wanpin 8203	*Hap2*	III
180	Wan 85-50-Fan3	*Hap3*	III
181	Wan 89193	*Hap3*	III
182	Wanpin 8337	*Hap1*	III
183	Wanpin 8410	*Hap1*	III
184	Huadong 6	*Hap2*	III
185	Siyang 117	*Hap2*	III
186	Sumai 3	*Hap2*	III
187	Wangmai 17	*Hap2*	III
188	Kang R16	*Hap2*	III
189	Ningmaizi 44	*Hap3*	III

190	Yangmai 158	*Hap1*	III
191	Huadong 10	*Hap1*	III
192	Ning 8343	*Hap1*	III
193	Ning 8537	*Hap3*	III
194	Ningmaizi 3	*Hap1*	III
195	Ningmaizi 13	*Hap1*	III
196	Ning 8924	*Hap2*	III
197	Ningmaizi 19	*Hap2*	III
198	Sujian 14	*Hap2*	III
199	Nanda 8910	*Hap3*	III
200	Nannong 2293xuan	*Hap1*	III
201	Liying 1	*Hap1*	III
202	Liying 5	*Hap3*	III
203	Zhongshan 9	*Hap2*	III
204	Jiulan	*Hap3*	III
205	Zhemai 1	*Hap3*	III
206	Limai 16	*Hap2*	III
207	Jiamai 25	*Hap2*	III
208	Nanzhongzao	*Hap2*	III
209	Zhemai 4	*Hap2*	III
210	Zhexuan 78-23	*Hap3*	III
211	Emai 6	*Hap2*	III
212	Jingzhou 2	*Hap2*	III
213	Exi 84-1031	*Hap2*	III
214	Exi 652	*Hap2*	III
215	Enmai 4	*Hap1*	III
216	E 811	*Hap2*	III
217	Yimai 1	*Hap2*	III
218	Yixi 102	*Hap2*	III
219	Xiangmai 8	*Hap2*	III
220	Xiangmai 12	*Hap2*	III
221	Zhongjiwan	*Hap2*	III
222	Xiang 791-2	*Hap2*	III
223	Zhongana 875	*Hap2*	III
224	Gan 162	*Hap2*	III
225	Hechang 45	*Hap2*	IV
226	Shuwan 8	*Hap1*	IV
227	Sichuan 51	*Hap1*	IV
228	Chuanmai 10	*Hap2*	IV
229	Chuanmai 19	*Hap2*	IV
230	Chuanmai 22	*Hap3*	IV
231	Yaanzao	*Hap3*	IV
232	Fan 6	*Hap3*	IV
233	Hongai 1	*Hap3*	IV

234	Xichangfanxiumai	*Hap1*	IV
235	Chuanyu 12	*Hap2*	IV
236	Mianyang 11	*Hap3*	IV
237	Mianyang 26	*Hap1*	IV
238	Chuan 83C-1001	*Hap2*	IV
239	Chuan 7911	*Hap2*	IV
240	Xichang 5548-9	*Hap2*	IV
241	Pan 86001-3	*Hap2*	IV
242	Pan 88080-3-1-1	*Hap2*	IV
243	Bimai 10	*Hap2*	IV
244	Bimai 13	*Hap2*	IV
245	Bimai 26	*Hap2*	IV
246	Xingzhuai 3	*Hap2*	IV
247	Qianjian 28	*Hap2*	IV
248	Guinong 10	*Hap2*	IV
249	Guinong Y13	*Hap2*	IV
250	Gui 775	*Hap2*	IV
251	Xingyingwu 3	*Hap2*	IV
252	Kenguia 1	*Hap2*	IV
253	Xingyi 4	*Hap2*	IV
254	Hemai 8052	*Hap2*	IV
255	Yunmai 28	*Hap2*	IV
256	Yunmai 29	*Hap3*	IV
257	Yunmai 33	*Hap2*	IV
258	Yunmai 34	*Hap2*	IV
259	Fengmai 11	*Hap2*	IV
260	Nanyuan 1	*Hap2*	IV
261	Jingmai 2	*Hap1*	IV
262	Pu 170	*Hap2*	IV
263	Dian 622-525-2	*Hap1*	IV
264	Dian 8613	*Hap1*	IV
265	Kangxiu 10	*Hap3*	V
266	Dixiuzao	*Hap2*	V
267	Jinmai 2148	*Hap2*	V
268	Longxi 35	*Hap2*	V
269	Fufan 904	*Hap3*	V
270	Yuanshan	*Hap3*	V
271	Taizhongxuan 2	*Hap2*	V
272	Taizhong 23	*Hap1*	V
273	Dongnong 101	*Hap1*	VI
274	Hezuo 3	*Hap2*	VI
275	Hei 78-1259	*Hap2*	VI
276	Heifu 84S1378	*Hap3*	VI
277	Jia 6268A-549	*Hap1*	VI

278	Jia 84-S437	*Hap2*	VI
279	Kefeng 3	*Hap2*	VI
280	Kelao 4	*Hap2*	VI
281	Kequn	*Hap2*	VI
282	Kenbei 1	*Hap1*	VI
283	Kenda 1	*Hap1*	VI
284	Kenhong 15	*Hap2*	VI
285	Long 79-9468	*Hap2*	VI
286	Longfumai 2	*Hap2*	VI
287	Longmai 18	*Hap2*	VI
288	Longmai 19	*Hap2*	VI
289	Xinkehan 9	*Hap2*	VI
290	Xinshuguang 1	*Hap2*	VI
291	Xinshuguang 6	*Hap1*	VI
292	Fengqiang 3	*Hap1*	VI
293	Jichun 1016	*Hap1*	VI
294	Xiaobingmai 33	*Hap2*	VI
295	Jinghong 5	*Hap1*	VII
296	Pinchun 14	*Hap1*	VII
297	Jiba 7529	*Hap2*	VII
298	Jichun 8055-1	*Hap3*	VII
299	Yanbei 8	*Hap2*	VII
300	Jinchun 3	*Hap3*	VII
301	Lianglaiyoubaipixiaomai	*Hap3*	VII
302	Bihongsui	*Hap2*	VII
303	Neimai 11	*Hap2*	VII
304	Ganmai 6	*Hap3*	VIII
305	Ganmai 8	*Hap1*	VIII
306	Longchun 7	*Hap1*	VIII
307	Dingxi 24	*Hap1*	VIII
308	Linnong 12	*Hap2*	VIII
309	Jinmai 4	*Hap2*	VIII
310	Wudu 5	*Hap1*	VIII
311	Longdong 1	*Hap2*	VIII
312	Xifeng 9	*Hap2*	VIII
313	Xifeng 10	*Hap2*	VIII
314	Qingfeng 1	*Hap2*	VIII
315	Zhangchun 9	*Hap1*	VIII
316	Ganmai 46	*Hap3*	VIII
317	Huining 5	*Hap2*	VIII
318	Huining 10	*Hap2*	VIII
319	Wuchun 1	*Hap2*	VIII
320	Jinmai 303	*Hap2*	VIII
321	Gan Tal21-10-2	*Hap2*	VIII

322	Gan 8221-1-1	*Hap2*	VIII
323	Gan 8358-2	*Hap1*	VIII
324	Hongtu	*Hap1*	VIII
325	Ningchun 4	*Hap2*	VIII
326	Shi 886	*Hap2*	VIII
327	Ning 87N2801	*Hap2*	VIII
328	Jian 72	*Hap2*	VIII
329	Xiangnong 3	*Hap3*	VIII
330	Gaoyuan 506	*Hap1*	VIII
331	Huzhuhong	*Hap2*	VIII
332	Qingchun 25	*Hap2*	VIII
333	Qingchun 28	*Hap2*	VIII
334	Gaoyuan 338	*Hap1*	VIII
335	Gaoyuan 602	*Hap2*	VIII
336	Zangdong 4	*Hap2*	IX
337	Rikaze 7	*Hap2*	IX
338	Rikaze 8	*Hap2*	IX
339	Rikaze 54	*Hap2*	IX
340	Xindong 2	*Hap2*	X
341	Changdong 5	*Hap1*	X
342	Kashi 1	*Hap2*	X
343	Kashibaipi	*Hap2*	X
344	Tuokexun 1	*Hap2*	X
345	Tuchun 6	*Hap2*	X
346	Xinchun 2	*Hap2*	X
347	Jiudong 2	*Hap2*	X
348	Yinong 8	*Hap1*	X

Supplementary Table 5. Marker/trait association analysis of *TaSPL14-7A* haplotypes in 348 modern cultivars (MC) grown in three environments.

**Traits**	**2002LY**		
	7A-Hap1	***7A-Hap2***	***7A-Hap3***
HD (d)	175.42±0.52a	177.16±0.39b	176.77±0.74ab
MD (d)	226.55±0.85a	226.15±0.52a	227.02±1.04a
SL (cm)	10.5±0.21a	10.67±0.15a	10.13±0.29a
SN	21.23±0.28a	21.14±0.18a	21.52±0.34a
PH (cm)	91.16±8.54ab	91.42±1.25a	98.12±3.11b
GN	52.41±1.13a	52.07±0.77a	54.28±1.39a
ETN	6.96±0.26a	7.08±0.18a	7.95±0.37b
TKW (g)	43.77±0.65A	43.27±0.45A	38.28±0.89B
KL (cm)	0.67±0.01A	0.68±0.00A	0.64±0.01B
KW (cm)	0.34±0.00a	0.34±0.00a	0.33±0.00a
KT (cm)	0.29±0.00a	0.29±0.00a	0.28±0.00a

Traits	2005LY		
	7A-Hap1	***7A-Hap2***	***7A-Hap3***
HD (d)	198.95±0.43a	199.47±0.28a	199.71±0.50a
MD (d)	237.07±0.44a	237.16±0.25a	237.4±0.50a
SL (cm)	9.16±0.21ab	9.34±0.11a	8.75±0.22b
SN	21.37±0.23a	21.28±0.14a	21.21±0.29a
PH (cm)	88.67±2.07a(A)	93.04±1.23a(AB)	99.12±2.75b(B)
GN	46.37±0.83a	47.54±0.61a	46.86±1.25a
ETN	8.57±0.34a	9.11±0.25a	9.34±0.47a
TKW (g)	40.7±0.78A	40.04±0.42A	35.84±0.95B
KL (cm)	0.69±0.01A	0.69±0.00A	0.66±0.01B
KW (cm)	0.33±0.00A	0.33±0.00A	0.32±0.00B
KT (cm)	0.30±0.00A	0.29±0.00B	0.29±0.00B
**Traits**	**2010SY**		
	7A-Hap1	***7A-Hap2***	***7A-Hap3***
HD (d)	217.45±0.38a	217.46±0.24a	218.27±0.47a
MD (d)	253.09±0.35a	253.16±0.22a	254.02±0.45a
SL (cm)	9.76±0.17a	10.08±0.11a	9.74±0.23a
SN	20.53±0.21a	20.75±0.14a	20.70±0.30a
PH (cm)	87.58±1.91a(A)	90.16±1.15a(AB)	95.57±2.32b(B)
GN	54.61±0.93a	53.42±0.65a	53.82±1.40a
ETN	11.70±0.52AB	11.34±0.22A	13.11±0.61B
TKW (g)	40.95±0.81A	40.7±0.39A	35.37±0.86B
KL (cm)	0.69±0.01A	0.69±0.00A	0.66±0.01B
KW (cm)	0.33±0.00A	0.32±0.00A	0.31±0.00B
KT (cm)	0.30±0.00a	0.30±0.00a	0.31±0.02a

HD, heading date; MD, maturity date; SL, spike length; SN, spikelet number per spike; PH, plant height; GN, grain number per spike; ETN, effective tiller number; TKW, thousand kernel weight; KL, kernel length; KW, kernel width; KT, kernel thickness. Data are means ± SE. Uppercase letters and lowercase letters indicate extremely significant ($P < 0.01$) and significant differences ($P < 0.05$) between haplotypes respectively.

Supplementary Table 6. Genotypes of 1,051 global accessions.

Number	Accession	*TaSPL14-7A* haplotype
**European varieties**		
1	AMADEUS	*Hap1*
2	BRILLANT	*Hap2*
3	CHRISTIAN	*Hap2*
4	DUCK	*Hap2*
5	ERLA KOLBEN	*Hap2*
6	FERDINAND	*Hap1*
7	HARRACH-6111	*Hap2*
8	HARRACH-6112	*Hap2*
9	LENTIA	*Hap2*
10	MULTIBRAUN	*Hap2*
11	NEUHOF 1	*Hap1*
12	OENUS	*Hap2*
13	PRIMUS	*Hap1*
14	PROBSTDORFER ACCORD	*Hap1*
15	PROBSTDORFER MARTIN	*Hap1*
16	PROBSTDORFER PERLO	*Hap2*
17	PROBSTDORFER POKAL	*Hap1*
18	PROTECTOR	
19	RINNER WINTERWEISEN	*Hap2*
20	STABIL	*Hap2*
21	SVENNO GRANNEN	*Hap2*
22	VERBESSERTER-STJOHANNER	*Hap1*
23	AROSO	*Hap1*
24	CAMA	*Hap2*
25	CELESTA	*Hap2*
26	CORA	*Hap2*
27	GABY	*Hap1*
28	HYBRIDE DU CENTENAIRE	*Hap2*
29	JASON	*Hap2*
30	JUFY I	*Hap2*
31	LEDA	*Hap2*
32	MARCO	*Hap2*
33	MARYSA	*Hap2*
34	MINA	*Hap2*
35	Odeon	*Hap2*
36	PANTER	*Hap2*
37	PHOEBUS	*Hap1*
38	PONY	*Hap2*
39	PRIMA	*Hap2*

40	PROFESSEUR DELOS	*Hap2*
41	RENVAL	*Hap2*
42	RUSTIQUE	*Hap2*
43	STELLA	*Hap2*
44	TRESOR_DE_GEMBLOUX	*Hap1*
45	BULGARIA301	*Hap1*
46	CARODEJKA'S'-	*Hap2*
47	COLOMBO	*Hap2*
48	GUSSENKA	*Hap1*
49	HEBROS	*Hap2*
50	IANTER	
51	IASEN	*Hap2*
52	KS414	*Hap2*
53	OAZIS	*Hap1*
54	PERLA 1	*Hap3*
55	PLISKA	*Hap2*
56	PLISKA	*Hap1*
57	PLOUDIN 1015	
58	PRIASPA	*Hap2*
59	RUSALKA	*Hap2*
60	SADOVO8	*Hap2*
61	SKITIA	*Hap1*
62	SLAVYANKA	
63	STEPNO	*Hap2*
64	TRAJANA	*Hap2*
65	UBILEI	*Hap2*
66	YANA	*Hap2*
67	ARDUS	*Hap2*
68	ARINA	*Hap2*
69	BERNINA	*Hap2*
70	BESSO	*Hap1*
71	Bretonnieres	*Hap1*
72	DADORA	*Hap2*
73	EIGER	*Hap2*
74	FERMO	*Hap2*
75	GRANAT	*Hap2*
76	JUNZI	*Hap1*
77	LITA	*Hap1*
78	Lona	*Hap1*
79	MONT-CALME	*Hap2*
80	PAILLY	*Hap2*
81	PROBELLE	*Hap1*
82	Rouge de la Venoge	*Hap1*
83	ROUGE DE SORAT	*Hap1*

84	ROUGE DE VAUMARCUS	*Hap2*
85	VUITEBOEUF	*Hap1*
86	Wagenburger	*Hap2*
87	ZENTA	*Hap1*
88	ASTA	*Hap1*
89	Blava	*Hap2*
90	BREA	*Hap2*
91	BRUTA	*Hap2*
92	DOBROVICKA	*Hap2*
93	HODONINSKA_OSINATKA	*Hap2*
94	KASTICKA OSINATKA	*Hap1*
95	Lada	*Hap2*
96	Linda	*Hap2*
97	Livia	*Hap1*
98	Maja	*Hap2*
99	Oska	*Hap1*
100	Pavlina	*Hap2*
101	Regina	*Hap2*
102	Saxana	*Hap1*
103	Senta	*Hap1*
104	SLOVENSKA INTENSIVNA	*Hap2*
105	Viginta	*Hap1*
106	Vlasta	*Hap2*
107	ZDAR	*Hap2*
108	ZIDLOCHOVICKA OSINATKA	*Hap1*
109	Zora	*Hap1*
110	Almus	*Hap1*
111	CARDOS	*Hap2*
112	CARIMULTI	*Hap1*
113	CARPO	*Hap2*
114	Combi	*Hap2*
115	CRiEWENER	*Hap2*
116	Fortschritt	*Hap2*
117	GARANT	*Hap1*
118	Heines Kolben	*Hap2*
119	Ibis	*Hap2*
120	KOLIBRI	*Hap1*
121	Mikon	*Hap2*
122	MIRAS	*Hap2*
123	Ordeal	*Hap2*
124	PROBAT	*Hap2*
125	RECORD	*Hap2*
126	Riebesel 47-51	*Hap2*
127	SIEGERLANDER	*Hap2*

128	Taras	*Hap2*
129	Trane	*Hap1*
130	TREND	*Hap2*
131	ARAGON 03	*Hap2*
132	ARGANDA	*Hap2*
133	AZULON	*Hap1*
134	BIVONA	*Hap2*
135	CABEZON DE GONI	*Hap2*
136	CABEZON_DE_VALDEGONI	*Hap2*
137	CAMPEADOR	*Hap2*
138	CANALEJA 46	*Hap2*
139	Candeal	*Hap1*
140	CARRION-PIRON	*Hap1*
141	CATALAN-COMPACTO	*Hap1*
142	COMPADRE	*Hap2*
143	JEJA DE VALENCIA	*Hap2*
144	MAIORCA	*Hap1*
145	MONTEJUICK	*Hap1*
146	PANE 14	*Hap1*
147	PANE10	*Hap1*
148	PANE-247	*Hap2*
149	TREMESINO MONTEROSA	*Hap2*
150	TREMESINO XINZO DE LIMIA	*Hap2*
151	VILLAVERDE DE TRUCIOS	*Hap1*
152	XEXA DEL VALL	*Hap1*
153	AN048-19	*Hap2*
154	ANTTI	*Hap2*
155	APU	*Hap1*
156	JO3021	*Hap1*
157	JOUKO	*Hap1*
158	JUVA	*Hap1*
159	KIMMO	*Hap1*
160	NISU	*Hap2*
161	Olympia	*Hap1*
162	SOPU 7	*Hap2*
163	Tammi	*Hap1*
164	ALIGRE	*Hap1*
165	ALLIES	*Hap1*
166	ARPEGE	*Hap1*
167	AZTEC	*Hap2*
168	BLE DES DOMES	*Hap2*
169	CAPITOLE	*Hap2*
170	CHAMPLEIN	*Hap1*
171	ELITE LEPEUPLE	*Hap1*

172	ETOILE DE_CHOISY	*Hap1*
173	EUREKA	*Hap2*
174	FLORENCE_AURORE	*Hap2*
175	GOYA	*Hap2*
176	HYBRIDE_DE_BERSEE	*Hap2*
177	HYBRIDE_DE_LA_PAIX	*Hap1*
178	HYBRIDE_DU_TRESOR	*Hap2*
179	ISENGRAIN	*Hap1*
180	JAPHET	*Hap1*
181	MAGALI BLONDEAU	*Hap1*
182	MARNE DESPREZ	*Hap2*
183	MELBOR	*Hap2*
184	NORD-DESPREZ	*Hap2*
185	PANIFOR	*Hap2*
186	SOISSONS	*Hap2*
187	TADEPI	*Hap2*
188	VILMORIN 53	*Hap1*
189	VILMORIN23	*Hap2*
190	VIRTUOSE	*Hap2*
191	YGA_BLONDEAU	*Hap2*
192	Banner	*Hap1*
193	BARON	*Hap1*
194	BENEFACTOR	*Hap2*
195	BRANDO	*Hap2*
196	BRIGADIER	*Hap2*
197	Brock	*Hap2*
198	GOLDENTROP	*Hap1*
199	HICKLING	*Hap1*
200	HICKLING BLANC DE MARS	*Hap2*
201	MALACCA	*Hap1*
202	Maris Beacon	*Hap2*
203	Maris Bilbo	*Hap2*
204	Maris Nimrod	*Hap1*
205	MARKET	*Hap1*
206	Norman	*Hap1*
207	SCHIREFF_SQUAREHEAD	*Hap2*
208	SHAMROCK	*Hap1*
209	SHANGO	*Hap2*
210	SHIREFF-BLANC-BARBU	*Hap2*
211	TORFIDA	*Hap2*
212	VICTOR	*Hap2*
213	Virtue	*Hap1*
214	ALFOLD	*Hap2*
215	Banatka	*Hap2*

216	BANATKA	*Hap1*
217	BETA BANKUTI	*Hap2*
218	ESTERHAZY	*Hap2*
219	FERTODI293	*Hap2*
220	GK Bank	*Hap2*
221	GK BENCE	*Hap2*
222	GK CSUROS	*Hap2*
223	GK Istvan	*Hap2*
224	GK ORZSE	*Hap2*
225	GK Szeged	*Hap1*
226	GK_BARNA	*Hap2*
227	GK_SAGVARI	*Hap2*
228	GKF2	*Hap2*
229	KARCAGI 522	*Hap1*
230	KOMPOLTI 169	*Hap2*
231	LOVASZPATONAI 407	*Hap2*
232	MARTONVASARI 23	*Hap2*
233	Mv Magvas	*Hap2*
234	MV SUMMA	*Hap2*
235	Mv Tamara	*Hap1*
236	Adria	*Hap1*
237	Aquila	*Hap1*
238	ARGELATO	*Hap2*
239	AUTONOMIA	*Hap2*
240	CAMPODORO	*Hap3*
241	CARLOTTA STRAMPELLI	*Hap1*
242	CERVARO	*Hap1*
243	ELIA	*Hap1*
244	Falchetto	*Hap2*
245	FLAMINIO	*Hap2*
246	Fortunato	*Hap2*
247	FRASSINETTO 4O5	*Hap1*
248	FUNO	*Hap1*
249	Funone	*Hap2*
250	GENTILE ROSSO	*Hap2*
251	Lario	*Hap1*
252	LEONE	*Hap1*
253	LONTRA	*Hap1*
254	Loreto	*Hap1*
255	Maestra	*Hap2*
256	MARA	*Hap1*
257	Pegaso	*Hap2*
258	BALDUS	*Hap1*
259	BERCY	*Hap1*

260	Cleo	*Hap1*
261	Donata	*Hap1*
262	DONG	*Hap2*
263	EMMA	*Hap2*
264	Flevina	*Hap2*
265	KNAL	*Hap1*
266	LOUVRE	*Hap1*
267	MAGNUS	*Hap1*
268	MANSHOLTS-WITTE	*Hap2*
269	MILDRESS	
270	ORCA	*Hap2*
271	PANG	*Hap2*
272	PLOF	*Hap2*
273	ROTONDE	*Hap2*
274	SAMBO	*Hap1*
275	Sarno	*Hap2*
276	STARING	*Hap2*
277	VADA	*Hap1*
278	VASCO	*Hap2*
279	VERSAILLES	
280	Banatka	*Hap2*
281	DANKOWSKA IDEALNA	*Hap2*
282	EKA NOWA	*Hap2*
283	GRANIATKA ZACHODNIA	*Hap2*
284	JANA	*Hap1*
285	KOMOROWSKA-POL	*Hap2*
286	KUTNOWIANKA	*Hap2*
287	LUNA	*Hap2*
288	MODRA	*Hap1*
289	OLZA	*Hap2*
290	OSTKA POPULARNA	*Hap2*
291	PANDA	*Hap2*
292	PODKOWIANKA	*Hap2*
293	POLANKA	*Hap1*
294	Przodownica	*Hap2*
295	ROZTOCKA	*Hap2*
296	Slazaczka	*Hap1*
297	Szelejewska	*Hap1*
298	WENEDA	*Hap1*
299	WYSOKOLITEWKA 218	*Hap2*
300	Wysokolitewka Sztywnosloma	*Hap2*
301	ZELAZNA	*Hap2*
302	ALMONSOR	*Hap2*
303	Amarelo de Barba Branca	*Hap2*

304	ELVAS 61P17	*Hap2*
305	ELVAS 62H12	*Hap2*
306	ELVAS 62H9	*Hap2*
307	ELVAS 63P16	*Hap1*
308	ELVAS 68H16	*Hap2*
309	ELVAS-60P24	*Hap2*
310	PADEIRA	*Hap2*
311	RIBEIRO INERME	*Hap2*
312	RIBEIRO~PI185706	*Hap2*
313	RIBEIRO~PI192624	*Hap1*
314	RIBEIRO~PI266901	*Hap1*
315	RUIVO	*Hap1*
316	TUA	*Hap1*
317	ALBOTA	*Hap2*
318	ARIESAN	*Hap2*
319	BUCOVINA	*Hap2*
320	CERES	*Hap2*
321	DOINA	*Hap2*
322	FAVORIT	*Hap2*
323	FLAMURA 80	*Hap1*
324	FUNDULEA 4	*Hap2*
325	FUNDULEA 262	*Hap2*
326	FUNDULEA133	*Hap1*
327	IULIA-ROM	*Hap1*
328	LOVRIN 10	*Hap2*
329	LOVRIN 13	*Hap1*
330	LOVRIN 29	*Hap2*
331	LOVRIN 32	*Hap2*
332	LOVRIN 34	*Hap2*
333	MAGURELE 7	*Hap2*
334	ODVOS 5	*Hap2*
335	POTAISSA	*Hap1*
336	SILVANA	*Hap1*
337	TRANSILVANIA1	*Hap2*
338	TURDA 195	*Hap2*
339	ALGOT	*Hap1*
340	AROS	*Hap2*
341	ATLE	*Hap2*
342	BANCO	*Hap1*
343	CROUSTY	*Hap1*
344	Diamant	*Hap2*
345	ERGO II	*Hap1*
346	FAGOTT	*Hap1*
347	IDUNA	*Hap2*

348	KUBB	*Hap1*
349	NORRE	*Hap2*
350	ORCANO	*Hap1*
351	Pansar II	*Hap1*
352	POMPE	*Hap2*
353	PONDUS	*Hap2*
354	Saffran	*Hap2*
355	SKANDIA	*Hap1*
356	SOLEIL	*Hap2*
357	Sunnan	*Hap1*
358	THULLE II	*Hap2*
359	TIMMO	*Hap2*
360	VIRTUS	*Hap1*
361	AGROUNIJA	*Hap2*
362	Banacanka 1	*Hap2*
363	Banatka	*Hap2*
364	BARANJKA	*Hap2*
365	BECEJKA	*Hap1*
366	CERVENA VROUSKA	*Hap2*
367	Dobro polje	*Hap2*
368	DUGOKLASA	*Hap1*
369	Dukat	*Hap2*
370	JARKA	*Hap1*
371	KRAJINKA	*Hap2*
372	LEPENICA	*Hap2*
373	NOVOSADSKA CRVENA	*Hap2*
374	OROVCANKA	*Hap2*
375	PANONIJA	*Hap2*
376	POSAVKA	*Hap3*
377	RADUSA	*Hap2*
378	RAVNICA	*Hap2*
379	Sava	*Hap2*
380	STIZANKA	*Hap2*
381	SUMADIJA	*Hap1*
382	Sutjeska	*Hap2*
383	Bourgeois	*Hap2*
384	Blausamliger Kolben	*Hap1*
**North American varieties**		
1	ZIMMERMAN	*Hap2*
2	Pride of Genesee	*Hap2*
3	Democrat	*Hap1*
4	Red Chief	*Hap3*
5	Gipsy	*Hap1*
6	Red Wave	*Hap1*

7	Power	*Hap2*
8	Sherman	*Hap1*
9	White Odessa	*Hap1*
10	Fulcaster	*Hap1*
11	Kanred	*Hap1*
12	Nebraska No. 28	*Hap1*
13	Minhardi	*Hap1*
14	Mediterranean	*Hap2*
15	Red May	*Hap2*
16	Imperial Amber	*Hap3*
17	Illini Chief	*Hap1*
18	Red Rock	*Hap1*
19	Rice	*Hap2*
20	Penquite	*Hap2*
21	Minturki	*Hap2*
22	Nebraska No. 60	*Hap2*
23	Blackhull	*Hap1*
24	Wisconsin Pedigree No. 2	*Hap2*
25	Forward	*Hap3*
26	Karmont	*Hap1*
27	Marquillo	*Hap3*
28	Ceres	*Hap2*
29	Iobred	
30	Newturk	*Hap1*
31	Tenmarq	*Hap1*
32	Yogo	*Hap2*
33	Kawvale	*Hap1*
34	Ilred	*Hap2*
35	Oro	*Hap1*
36	Purkof	*Hap1*
37	Red Indian	*Hap2*
38	Early Blackhull	*Hap2*
39	Nabob	*Hap2*
40	Cheyenne	*Hap1*
41	Quivira	*Hap1*
42	Thatcher	*Hap2*
43	Rio	*Hap1*
44	Golden	*Hap2*
45	Relief	*Hap1*
46	Nebred	*Hap1*
47	Wabash	*Hap1*
48	Ioturk	*Hap2*
49	Lemhi	*Hap1*
50	Pilot	*Hap1*

51	Marmin	*Hap1*
52	Enid	*Hap2*
53	Baldrock	*Hap2*
54	Utah Kanred	*Hap1*
55	Pawnee	*Hap1*
56	Comanche	*Hap2*
57	Thorne	*Hap2*
58	Marfed	*Hap2*
59	Mida	*Hap1*
60	Cadet	*Hap1*
61	Westar	*Hap1*
62	Ponca	*Hap2*
63	Triumph	*Hap1*
64	Kiowa	*Hap1*
65	Minter	*Hap1*
66	Sioux	*Hap1*
67	Quanah	*Hap3*
68	Blackhawk	*Hap1*
69	Chancellor	*Hap1*
70	Austin	*Hap2*
71	Brevor	*Hap2*
72	Ramona 50	*Hap2*
73	Nured	*Hap1*
74	Taylor	*Hap2*
75	Russell	
76	Lee	*Hap2*
77	Concho	*Hap1*
78	Bison	*Hap1*
79	Butler	*Hap2*
80	Seneca	*Hap2*
81	Atlas 50	*Hap1*
82	LaPorte	*Hap2*
83	Atlas 66	*Hap2*
84	Coker 47-27	*Hap1*
85	Genesee	*Hap1*
86	Saline	
87	Vermillion	*Hap1*
88	Tayland	*Hap2*
89	Westmont	*Hap1*
90	Itana	*Hap1*
91	Centana	*Hap2*
92	Tascosa	*Hap2*
93	Onas 53	*Hap1*
94	Dual	*Hap1*

95	Conley	*Hap1*
96	Racine	*Hap1*
97	Warrior	*Hap2*
98	Taylor 49	*Hap1*
99	Monon	*Hap1*
100	Georgia 1123	
101	Wakeland	*Hap1*
102	Ace	*Hap2*
103	Delmar	*Hap1*
104	Gaines	*Hap1*
105	Avon	*Hap1*
106	Reed	*Hap1*
107	Hume	*Hap1*
108	Gage	*Hap1*
109	Caddo	*Hap1*
110	Scout	*Hap1*
111	Triumph 64	*Hap2*
112	Sturdy	*Hap1*
113	Moro	*Hap2*
114	Polk	*Hap1*
115	Timwin	*Hap2*
116	Wanser	*Hap1*
117	Guide	*Hap1*
118	Fletcher	*Hap1*
119	Era	*Hap1*
120	Scout 66	*Hap2*
121	Trader	*Hap1*
122	Trapper	*Hap2*
123	Kenosha	*Hap2*
124	Yorkstar	*Hap2*
125	Blueboy	*Hap1*
126	Scoutland	*Hap1*
127	Pronto	*Hap1*
128	Logan	*Hap1*
129	Ionia	*Hap2*
130	Paha	*Hap2*
131	Caprock	*Hap2*
132	McDermid	*Hap2*
133	Bridger	*Hap2*
134	Chanute	*Hap2*
135	Satanta	*Hap2*
136	Yukon	*Hap2*
137	Luke	*Hap2*
138	Centurk	*Hap1*

139	Bounty 208	*Hap1*
140	Shortana	*Hap2*
141	Blueboy II	*Hap1*
142	Anza	*Hap1*
143	Potomac	*Hap1*
144	Ranger	*Hap1*
145	Sprague	*Hap1*
146	Baca	*Hap1*
147	Oasis	*Hap3*
148	Hiplains	*Hap1*
149	Buckskin	*Hap1*
150	Cloud	
151	Sage	*Hap1*
152	Ticonderoga	*Hap1*
153	Gent	*Hap1*
154	Lancota	*Hap1*
155	Newana	*Hap2*
156	Roughrider	*Hap2*
157	Agate	*Hap1*
158	Rall	*Hap2*
159	Larned	*Hap2*
160	Arbon	*Hap1*
161	Tyee	*Hap1*
162	TAM 105	*Hap2*
163	Pike	*Hap1*
164	Tyler	*Hap2*
165	Arkan	*Hap1*
166	TAM 107	*Hap1*
167	TAM 108	*Hap2*
168	Kitt	*Hap2*
169	Huron	*Hap1*
170	Preston	*Hap2*
171	Dawson	*Hap1*
172	Marquis	*Hap1*
173	Prelude	*Hap1*
174	Pioneer	*Hap1*
175	White Fife	*Hap1*
176	Stanley	*Hap1*
177	Kitchener	*Hap1*
178	Early Red Fife	*Hap1*
179	Ruby	*Hap1*
180	Nebraska No. 6	*Hap1*
181	Red Bobs	*Hap1*
182	Sea Island	*Hap1*

183	Kharkov MC22	*Hap2*
184	Oregon Zimmerman	*Hap2*
185	Supreme	*Hap2*
186	Reward	*Hap1*
187	Renfrew	*Hap1*
188	Axminster	*Hap1*
189	Escondido	*Hap2*
190	Ramona	*Hap2*
191	Arco	*Hap1*
192	Hard Federation 31	*Hap2*
193	Berkeley Rock	*Hap1*
194	Powerclub	*Hap1*
195	Whiteman	*Hap1*
196	Clarkan	*Hap1*
197	Eagle Chief	*Hap2*
198	Hyper	*Hap2*
199	Marvel	*Hap1*
200	Montana King	*Hap1*
201	Poso	*Hap1*
202	Redhart	*Hap2*
203	Missouri Valley	*Hap3*
204	V.P.I. 131	
205	V.P.I. 112	*Hap1*
206	Hood	*Hap1*
207	Comet	*Hap1*
208	Coronation	*Hap1*
209	Kruse	*Hap1*
210	Redhull	*Hap1*
211	Genro	*Hap1*
212	Erect	*Hap1*
213	Hymar	*Hap2*
214	Renown	*Hap1*
215	Apex	*Hap1*
216	Rival	*Hap1*
217	Regent	*Hap1*
218	Orfed	*Hap1*
219	Fairfield	*Hap1*
220	Sanford	*Hap1*
221	Newthatch	*Hap1*
222	Cascade	*Hap1*
223	Rescue	*Hap1*
224	Saunders	*Hap1*
225	Redman	*Hap1*
226	Selkirk	*Hap1*

227	Chinook	*Hap1*
228	Kenhi	*Hap2*
229	Pembina	*Hap2*
230	Cypress	*Hap1*
231	Canthatch	*Hap2*
232	Lake	*Hap1*
233	Winalta	*Hap2*
234	Manitou	*Hap1*
235	Talbot	*Hap2*
236	Park	*Hap2*
237	Neepawa	*Hap1*
238	McNair 1813	*Hap1*
239	Sundance	*Hap1*
240	Inia 66R	*Hap2*
241	Canuck	*Hap2*
242	Yecora Rojo	
243	Portola	*Hap1*
244	Downy	
245	Sinton	
246	Rosen	
247	Shasta	*Hap1*
248	Roland	*Hap1*
249	Payne	*Hap2*
250	Omega 78	*Hap1*
251	Bennett	*Hap1*
252	Centurk 78	*Hap1*
253	Maverick	*Hap1*
254	Texred	*Hap2*
255	Norstar	*Hap2*
256	Dirkwin	*Hap2*
257	Powell	*Hap1*
258	Nelson	*Hap2*
259	Purcell	*Hap2*
260	James	*Hap1*
261	Rose	*Hap1*
262	Rita	*Hap2*
263	Dawn	*Hap1*
264	Nell	*Hap2*
265	TAM 106	*Hap2*
266	Pondera	*Hap2*
267	Frankenmuth	*Hap1*
268	Augusta	*Hap1*
269	Redwin	*Hap1*
270	Manning	*Hap1*

271	Crestone	*Hap2*
272	Sterling	*Hap2*
273	Neeley	*Hap1*
274	Stacy	*Hap2*
275	Wings	*Hap1*
276	Tamex	*Hap2*
277	Mit	*Hap2*
278	Caldwell	*Hap1*
279	Auburn	*Hap1*
280	Wheeler	*Hap2*
281	Winridge	*Hap1*
282	McKay	*Hap1*
283	Owens	*Hap1*
284	Lewjain	*Hap2*
285	Tres	*Hap2*
286	Marshall	*Hap2*
287	Guard	*Hap1*
288	Centa	*Hap2*
289	Severn	*Hap2*
290	Crew	*Hap2*
291	Massey	*Hap2*
292	Hill 81	*Hap1*
293	Phoenix	*Hap1*
294	Brule	*Hap1*
295	Treasure	*Hap1*
296	Wheaton	*Hap1*
297	Fillmore	*Hap1*
298	Scotty	*Hap2*
299	Bradford	*Hap2*
300	Susquehanna	*Hap1*
301	Centura	*Hap1*
302	Colt	*Hap1*
303	Agassiz	*Hap1*
304	Compton	*Hap2*
305	Saluda	*Hap2*
306	Adena	*Hap1*
307	Glenman	*Hap1*
308	Siouxland	*Hap1*
309	Cody	*Hap1*
310	Chisholm	*Hap1*
311	Bliss	*Hap2*
312	Dusty	*Hap2*
313	Hillsdale	*Hap2*
314	Ute	*Hap1*

315	Adder	*Hap2*
316	Cree	*Hap1*
317	Norwin	*Hap2*
318	Prospect	*Hap2*
319	Shield	*Hap1*
320	Tadinia	*Hap1*
321	Becker	*Hap2*
322	Batum	*Hap1*
323	Leader	*Hap1*
324	Blanca	*Hap1*
325	Copper	*Hap1*
326	Redland	*Hap2*
327	Century	*Hap1*
328	Cardinal	*Hap1*
329	Geneva	*Hap1*
330	Dodge	*Hap1*
331	Norkan	*Hap1*
332	Dynasty	*Hap1*
333	Williams	*Hap2*
334	Lancer	*Hap2*
335	GR855	*Hap1*
336	GR863	*Hap2*
337	GR860	*Hap2*
338	Seward	*Hap1*
339	Survivor	*Hap2*
340	Madsen	*Hap1*
341	Hyak	*Hap1*
342	Collin	*Hap2*
343	Andrews	*Hap2*
344	Blizzard	*Hap1*
345	Clark	*Hap2*
346	Oveson	*Hap2*
347	GR876	*Hap2*
348	Keiser	*Hap2*
349	Tiber	*Hap1*
350	Arapahoe	*Hap2*
351	Laura	*Hap2*
352	Marberg	*Hap2*
353	Karl	*Hap2*
354	Minnpro	*Hap2*
355	Vance	*Hap2*
356	2548	*Hap1*
357	2555	*Hap1*
358	Buchanan	*Hap1*

359	Eltan	*Hap2*
360	Kmor	*Hap1*
361	Centennial	*Hap2*
362	SWS-52	*Hap1*
363	Georgia 100	*Hap2*
364	Sharp	*Hap1*
365	Rely	*Hap1*
366	Rawhide	
367	Vandal	*Hap1*
368	Wakefield	*Hap1*
369	Madison	*Hap1*
370	Verne	*Hap1*
371	Hi-Line	*Hap1*
372	Howell	*Hap1*
373	McNair 1003	*Hap2*
374	TAM 109	*Hap2*
375	Promontory	*Hap2*
376	Excel	*Hap2*
377	Fairview	*Hap1*
378	Rod	*Hap2*
379	Hoff	*Hap1*
380	Gene	*Hap1*
381	AC Reed	*Hap2*
382	GA-Gore	*Hap1*
383	GA-Andy	*Hap1*
384	GR915	*Hap1*
385	Vista	*Hap1*
386	Erhardt	*Hap2*
387	Jules	*Hap2*
388	AC Taber	*Hap1*
389	Alliance	*Hap1*
390	McNeal	*Hap1*
391	TAM-200	*Hap1*
392	TAM-201	*Hap1*
393	Sylvan	*Hap1*
394	Lambert	*Hap2*
395	AC Michael	*Hap2*
396	Akron	*Hap2*
397	Halt	*Hap2*
398	Judith	*Hap1*
399	Niobrara	*Hap1*
400	Nekota	*Hap2*
401	Clemson 201	*Hap2*
402	IL 84-4046	*Hap2*

403	IL 85-3132-1	*Hap2*
404	Finley	*Hap2*
405	Hiller	*Hap1*
406	Hazen	*Hap2*
407	USU-Apogee	
408	Glory	*Hap2*
409	Whitebird	*Hap1*
410	Pomerelle	*Hap2*
411	Pronghorn	*Hap1*
412	AC Barrie	*Hap2*
413	AC Karma	*Hap1*
414	Rampart	*Hap2*
415	McGuire	*Hap2*
416	Vanguard	*Hap2*
417	Coda	*Hap2*
418	Utah-100	*Hap1*
419	NY Batavia	*Hap1*
420	Cayuga	*Hap1*
421	BacUp	*Hap1*
422	Windstar	*Hap1*
423	Boundary	*Hap2*
424	Jefferson	*Hap2*
425	AC Nanda	*Hap1*
426	AC Phil	*Hap1*
427	Yumar	*Hap1*
428	Prowers	*Hap1*
429	Prairie Red	*Hap1*
430	Wesley	*Hap1*
431	Culver	*Hap1*
432	Edwin	*Hap2*
433	Hayden	*Hap2*
434	Harding	*Hap2*
435	Caledonia	
436	KY 86C-61-8	*Hap1*
437	Prowers 99	*Hap2*
438	Cougar	*Hap1*
439	Millennium	*Hap1*
440	Rick	*Hap1*
441	Wynne	*Hap1*
442	Jubilee	*Hap1*
443	Lolo	*Hap1*
444	Iona	*Hap2*
445	Wahoo	*Hap1*
446	NuSky	*Hap1*

447	Avalanche	*Hap1*
448	Finch	*Hap2*
449	Intrada	*Hap1*
450	Deloris	*Hap2*
451	Ok101	*Hap1*
452	Ankor	*Hap1*
453	Richland	*Hap2*
454	Jerry	*Hap2*
455	Goodstreak	
456	Harry	*Hap1*
457	Ok102	*Hap1*
458	Hollis	*Hap1*
459	AC Andrew	*Hap2*
460	Antelope	*Hap2*
461	Arrowsmith	*Hap1*
462	AR 910	*Hap2*
463	Hatcher	*Hap1*
464	Hallam	*Hap1*
465	Choptank	*Hap1*
466	Juniper	*Hap1*
467	UI Darwin	*Hap1*
468	Paul	*Hap1*
469	Allegiance	*Hap1*
470	Cataldo	*Hap1*
471	UI Winchester	*Hap1*
472	Bess	*Hap1*
473	Chesapeake	*Hap1*
474	NE01643	*Hap1*
475	Mace	*Hap2*
476	Camelot	*Hap2*
477	Willow Creek	*Hap1*
478	Alson	*Hap1*
479	Steel-ND	*Hap1*
480	Faller	*Hap2*

Former USSR varieties

1	L21	*Hap2*
2	L28	*Hap3*
3	Ф2а	*Hap2*
4	Ф3а	*Hap1*
5	G7	*Hap3*
6	G14	*Hap3*
7	B55	*Hap2*
8	D35	*Hap3*
9	Ак-Урук	*Hap2*

10	Чуионuозюахоп	*Hap3*
11	Zунюudахапuau	*Hap3*
12	Lvrin 37	*Hap2*
13	Vilmorin 30	*Hap1*
14	Волгоградская	*Hap2*
15	Bencubbin/s	*Hap2*
16	Copт-2	*Hap1*
17	Зерноградкая	*Hap2*
18	Зимородок	*Hap1*
19	Багра	*Hap1*
20	Багра	*Hap2*
21	Казанская 84	*Hap2*
22	Сая 95009	*Hap1*
23	Danchi Komugi	*Hap2*
24	Gabo	*Hap2*
25	Gabo-2	*Hap2*
26	Gabo-3	*Hap2*
27	Ellysee/s	*Hap2*
28	Эритроспермум 7291	*Hap2*
29	Тарасовская 61	*Hap2*
30	Куйбышевка	*Hap2*
31	Черноземка 153	*Hap2*
32	Югтина	*Hap1*
33	A33	*Hap2*
34	Багра-9	*Hap2*
35	Copт-1	*Hap2*
36	Erytrospermum 81	*Hap2*
37	Безостая 2	*Hap2*
38	Top/s	*Hap1*
39	Wim	*Hap2*
40	Danchi Komugi/t	*Hap3*
41	Лютесценс 329	*Hap2*
42	Landrace	*Hap2*
43	Landrace	*Hap1*
44	Bencubbin/wt	*Hap2*
45	Gabo	*Hap2*
46	Донская юбилейная	*Hap2*
47	Сибирская Нива	*Hap2*
48	Даха	*Hap2*
49	Бугдай	*Hap2*
50	Банаткя	*Hap3*
51	Gaba-1	*Hap2*
52	Крымская.обл.	*Hap2*
53	Сандомирка	*Hap2*

54	Lvrin 36	*Hap2*
55	Vilmorin 30	*Hap2*
56	Волгоградская 84	*Hap2*
57	Краснодарская 39	*Hap2*
58	Мироновская 28	*Hap2*
59	Сандомирка	*Hap2*
60	A06/w	*Hap2*
61	Анвхой11/s	*Hap2*
62	Local	*Hap1*
63	Local	
64	Kavkaz	
65	Ablaca	
66	Wheaton	*Hap1*
67	Прпобокая	*Hap2*
68	Cabezorro Ⅲ	*Hap1*
69	Liviongston	*Hap2*
70	E68	*Hap2*
71	S5	*Hap3*
72	H3(a)1	*Hap1*
73	Саратовская 51	*Hap2*
74	Боливиа	*Hap1*
75	Бурятская	*Hap1*
76	Erythrospermum 59/r	*Hap1*
77	Norm	*Hap1*
78	Yuriya 79	*Hap2*
79	Red River	*Hap2*
80	Ablaca-3	*Hap2*
81	Wheaton-1	*Hap2*
82	Yuriya 80	*Hap2*
83	Fortuna/t	*Hap1*
**CIMMYT varieties**		
1	YAQUI 48	
2	LERMA 50	*Hap2*
3	GABO 54	*Hap2*
4	PENJAMO T 62	*Hap2*
5	PITIC S62	*Hap2*
6	NADADORES M 63	*Hap2*
7	LERMA ROJO 64	*Hap3*
8	SONORA 64	*Hap2*
9	INIA F 66	*Hap2*
10	SIETE CERROS T66	*Hap2*
11	SONALIKA	
12	YECORA F 70	*Hap2*
13	MARCOS JUAREZ INTA	

14	ANZA	*Hap2*
15	JUPATECO F 73	*Hap2*
16	NACOZARI F 76	*Hap2*
17	PAVON F 76	*Hap1*
18	CIANO T 79	*Hap2*
19	SERI M 82	*Hap2*
20	PUNJAB 85	*Hap2*
21	DON ERNESTO INTA	*Hap2*
22	BACANORA T 88	*Hap2*
23	CUMPAS T 88	*Hap2*
24	NESSER	*Hap2*
25	BAVIACORA M 92	*Hap2*
26	SUPER KAUZ	*Hap2*
27	MISR 1	*Hap2*
28	EMBRAPA 16	*Hap1*
29	KINGBIRD #1	*Hap1*
30	THELIN#2/TUKURU	*Hap2*
31	BABAX/LR42	*Hap2*
32	ND643/2*WBLL1	*Hap2*
33	ND643/2*WBLL1	*Hap2*
34	DANPHE #1	*Hap2*
35	PICAFLOR #1	*Hap2*
36	YANAC/3/PARULA	*Hap1*
37	SHANGHAI#7	*Hap2*
38	QUAIU	*Hap2*
39	PARULA/2 PASTOR	*Hap2*
40	NELOKI	*Hap2*
41	HUW234+LR34/PRINIA	*Hap1*
42	WHEATEAR/SOKOLL	*Hap1*
43	FRET2*2/4/SONOITA F	*Hap2*
44	PBW343*2/KUKUNA	*Hap2*
45	WEEBILL1*2/BRAM	*Hap1*
46	ALTAR 84/AE.SQUARROSA	*Hap1*
47	UP2338*2/4/SONOITA	*Hap1*
48	PARUS/FRANCOLIN #1	*Hap2*
49	PFAU/SERI.1B//AMADINA	*Hap1*
50	NACOZARI F 76	*Hap2*
51	NACOZARI F 76	*Hap2*
52	ALTAR 84/AE.SQUARROSA	*Hap2*
53	PBW343	*Hap2*
**Australian varieties**		
1	Crusades	*Hap2*
2	Lincolia	*Hap2*
3	Dakota	*Hap2*

4	Bowerbird	*Hap2*
5	Baxter	*Hap2*
6	Wylah	*Hap2*
7	Wilgoyne	*Hap2*
8	Westonia	*Hap2*
9	Ventura	*Hap2*
10	Tasman	*Hap2*
11	Tailorbird	*Hap2*
12	Sunyale	*Hap2*
13	Sunsoft 98	*Hap2*
14	Sunlin	*Hap2*
15	Sunfield	*Hap2*
16	Suneco	*Hap2*
17	Sunbri	*Hap2*
18	Ruby	*Hap2*
19	Sapphire	*Hap2*
20	Petrie	*Hap2*
21	Rubric	*Hap1*
22	Roselia	*Hap2*
23	Perenjori	*Hap2*
24	Mitre	*Hap2*
25	Lorikeet	*Hap2*
26	Leichhardt	*Hap2*
27	Lang	*Hap2*
28	Kukri	*Hap2*
29	Krichauff	*Hap1*
30	Kennedy	*Hap2*
31	Janz	*Hap2*
32	Giles	*Hap2*
33	Prame	*Hap1*
34	Ega Gregory	*Hap2*
35	Ega Binnie Rock	*Hap2*
36	Drysdale	*Hap2*
37	Diamondbird	*Hap2*
38	Cunningham	*Hap2*
39	Cunderdin	*Hap2*
40	Cook	*Hap2*
41	Chara	*Hap2*
42	Casades	*Hap2*
43	Carnamah	*Hap2*
44	Carinya	*Hap2*
45	Camm	*Hap2*
46	Calingiri	*Hap2*
47	Cadoux	*Hap2*

48	Barks	*Hap2*
49	Arrino	*Hap2*
50	Annuello	*Hap2*
51	Amery	*Hap2*
